# Supplementary material for: Distance, difference in altitude and socioeconomic determinants of utilisation of maternal and child health services in Ethiopia: a geographic and multilevel modelling analysis
Source: BMJ Open. 2021 Feb 18;11(2):e042095. doi: 10.1136/bmjopen-2020-042095 (PMC7896622; doi:10.1136/bmjopen-2020-042095)
Supplement: Author's manuscript [file bmjopen-2020-042095.draft_revisions.pdf]

BMJ Open

BMJ Open is committed to open peer review. As part of this commitment we make the peer review history of every article we publish publicly available.

When an article is published we post the peer reviewers' comments and the authors' responses online. We also post the versions of the paper that were used during peer review. These are the versions that the peer review comments apply to.

The versions of the paper that follow are the versions that were submitted during the peer review process. They are not the versions of record or the final published versions. They should not be cited or distributed as the published version of this manuscript.

BMJ Open is an open access journal and the full, final, typeset and author-corrected version of record of the manuscript is available on our site with no access controls, subscription charges or pay-per-view fees (http://bmjopen.bmj.com).

If you have any questions on BMJ Open's open peer review process please email info.bmjopen@bmj.com

BMJ Open

Distance, difference in altitude, and socio-economic determinants of utilization of maternal and child health services in Ethiopia

Journal:	*BMJ Open*
Manuscript ID	bmjopen-2020-042095
Article Type:	Original research
Date Submitted by the Author:	26-Jun-2020
Complete List of Authors:	Defar, Atkure ; Ethiopian Public Health Institute; University of Gondar College of Medicine and Health Sciences, Institute of Public Health, Department of Epidemiology and Biostatistics<br>Okwaraji, Yemisrach; London School of Hygiene and Tropical Medicine Faculty of Infectious and Tropical Diseases, Department of Disease Control; Ethiopian Public Health Institute<br>Tigabu, Zemene; University of Gondar College of Medicine and Health Sciences, Department of Paediatrics and Child Health<br>Persson, Lars; London School of Hygiene and Tropical Medicine Faculty of Infectious and Tropical Diseases, Department of Disease Control; Ethiopian Public Health Institute<br>Alemu, Kassahun ; University of Gondar College of Medicine and Health Sciences, Institute of Public Health, Department of Epidemiology and Biostatistics
Keywords:	HEALTH SERVICES ADMINISTRATION & MANAGEMENT, Community child health < PAEDIATRICS, PUBLIC HEALTH

SCHOLARONE™
Manuscripts

I, the Submitting Author has the right to grant and does grant on behalf of all authors of the Work (as defined in the below author licence), an exclusive licence and/or a non-exclusive licence for contributions from authors who are: i) UK Crown employees; ii) where BMJ has agreed a CC-BY licence shall apply, and/or iii) in accordance with the terms applicable for US Federal Government officers or employees acting as part of their official duties; on a worldwide, perpetual, irrevocable, royalty-free basis to BMJ Publishing Group Ltd ("BMJ") its licensees and where the relevant Journal is co-owned by BMJ to the co-owners of the Journal, to publish the Work in this journal and any other BMJ products and to exploit all rights, as set out in our licence.

The Submitting Author accepts and understands that any supply made under these terms is made by BMJ to the Submitting Author unless you are acting as an employee on behalf of your employer or a postgraduate student of an affiliated institution which is paying any applicable article publishing charge ("APC") for Open Access articles. Where the Submitting Author wishes to make the Work available on an Open Access basis (and intends to pay the relevant APC), the terms of reuse of such Open Access shall be governed by a Creative Commons licence – details of these licences and which Creative Commons licence will apply to this Work are set out in our licence referred to above.

Other than as permitted in any relevant BMJ Author's Self Archiving Policies, I confirm this Work has not been accepted for publication elsewhere, is not being considered for publication elsewhere and does not duplicate material already published. I confirm all authors consent to publication of this Work and authorise the granting of this licence.

For peer review only

Distance, difference in altitude, and socio-economic determinants of utilization of maternal and child health services in Ethiopia

Atkure Defar[1, 4*], Yemisrach B. Okwaraji[1, 3], Zemene Tigabu[2], Lars Åke Persson[1, 3], Kassahun Alemu[4]

*Corresponding author:

Atkure Defar, MPH, MBA, Email: atkuredefar@gmail.com

ORCID, https://orcid.org/0000-0001-9435-2135

Co-authors:

Yemisrach Okwaraji, PhD, Email, yemisrach.okwaraji@lshtm.ac.uk

ORCID, https://orcid.org/0000-0002-4541-2856

Zemene Tigabu MD/Paediatrician, Email: zemene.tigabu@gmail.com

ORCID, https://orcid.org/0000-0001-6040-032X

Prof. Lars Åke Persson; MD, PhD, Email: Lars.Persson@lshtm.ac.uk

ORCID, https://orcid.org/0000-0003-0710-7954

Kassahun Alemu Gelaye, PhD, Email: kassalemu@gmail.com

ORCID, https://orcid.org/0000-0002-8360-0292

Affiliation

[1]Ethiopian Public Health Institute, Addis Ababa, Ethiopia

[2]Department of Paediatrics and Child Health, School of Medicine, College of Medicine and Health Sciences, University of Gondar, Gondar, Ethiopia

[3]London School of Hygiene and Tropical Medicine, London, UK

[4]Department of Epidemiology and Biostatistics, Institute of Public Health, College of Medicine and Health Sciences, University of Gondar, Gondar, Ethiopia

Word count 3372

For peer review only

ABSTRACT

Background In the strive towards universal health coverage, it is essential to reduce inequities in the utilization of health services. We assessed whether geographic distance and difference in altitude between home to health facility and household socio-economic group were associated with utilization of maternal and child health services in rural Ethiopia.

Methods A household and health facility surveys was conducted from December 2018 to February 2019 in 46 districts of four Ethiopian regions. A total of 11,877 women aged 13-49 years and 5,786 children aged 2-59 months were included. The outcomes were four or more antenatal care visits, facility delivery, full child immunization, and utilization of health services for sick children. A multilevel analysis was carried out with adjustments for potential confounding factors.

Results Overall, 39% (95%CI: 35-42) women had attended four or more antenatal care visits, and 55% (95%CI: 51-58) delivered at health facilities. One in three (36%, 95%CI: 33-39) of children had received full-immunizations and 35% (95%CI: 31-39) of sick children used health services. A long distance (AOR=0.57; 95% CI 0.34-0.96), and larger difference in altitude (AOR=0.34; 95% CI 0.19-0.59) were associated with fewer facility deliveries. Larger difference in altitude was associated with a lower proportion of antenatal care visits (AOR=0.46; 95%CI: 0.29-0.74). A higher wealth index was associated with a higher proportion of antenatal care visits (AOR=1.67; 95%CI: 1.02-2.75) and health facility deliveries (AOR=2.11; 95%CI: 2.11-6.48). There was no association between distance, difference in altitude, or wealth index and children being fully immunized or seeking care when they were sick.

Conclusion Achieving universal access to maternal and child health services will require not only strategies to increase coverage but also targeted efforts to address the geographic and socio-economic differentials in care utilization, especially for maternal health.

Keywords: Distance, Altitude, Antenatal care, Facility delivery, Immunization coverage, Sick child services utilization, Ethiopia

For peer review only

Article summary

Strength and limitation of this study

- The sample for this study was drawn from intervention and comparison areas for the evaluation of the Optimization of the Health Extension Program intervention, which was implemented in selected districts of the four most populous Ethiopian regions (15). Thus, the results may not be generalizable to the population at country level. We have, however, reasons to believe that the selected districts are typical for agrarian areas of these major Ethiopian regions.
- We assessed geographic and social equity across a continuum of maternal and child healthcare, i.e., antenatal care visits, facility delivery, immunization, and utilization of health services for sick children. This breadth allowed us to compare the associations between determinants and care utilization in different phases of this continuum. Previously published studies have focused on one or a few components of care utilization (38,39). In cross-sectional studies like this, there is a risk of biased reporting of care utilization. To reduce that risk, we limited the maternal services indicators to the last 12 months. The immunization coverage adhered to the methodology of the Demographic and Health Surveys, including children aged 12-23 months. Similarly, we followed the common practice of limiting common childhood illnesses and care seeking to a two-weeks recall.
- We assessed the straight-line distance between home and the nearest health facility. In reality, it may not be possible to travel in a straight line to a specific location and the route from home to the nearest health facility may be longer than the straight line as one has to avoid obstacles such as a river or a steep slope. In such cases, using cost-distance measurement may have given more realistic distance measurements. Some home-to-facility distance data were missing, especially regarding the maternal indicators, i.e., the distance from home to the health center. The reason was missing GPS coordinates of the health center the family was supposed to use. We have no reason to believe that these omissions introduced any biased estimates. We added the difference in altitude as a potentially important dimension in care utilization. In Ethiopia, with its mountains and valleys,

For peer review only - http://bmjopen.bmj.com/site/about/guidelines.xhtml

the difference in altitude expresses some of the time and exact distance by road or path from home to the health facility.

- We assessed altitude by GPS data that may not always be accurate. However, the error is usually of little importance; in the range from 10 to 20 meters. The socio-economic status was based on assets and characteristics of the household in the way that is practiced in the Demographic and Health Surveys. This characterization represents accumulated wealth and resources but does not directly represent the economic strength of the household to cover costs for care-seeking. Given the relative imprecision of the included determinants, we categorized these data into tertiles.
- We used multi-level regression modeling that included the determinants, the outcome, and potential confounding factors at the cluster, household, and individual levels. The regression models accounted for confounding and clustering effects.

What is already known on the subject and What this study adds

- In most low-income countries, utilization of maternal and child health services vary by geographic distance from home to health facilities and household wealth categories
- In this study from rural areas in four Ethiopian regions there were geographic and wealth-related inequities in facility-based deliveries, and difference in altitude between home and health facility was linked to coverage of antenatal care and facility delivery
- Immunization coverage and use of services for the sick child were on a low level but did not differ by geography and household wealth
- Efforts towards universal health coverage should include targeted efforts to address the geographic and social differentials in care utilization

INTRODUCTION

The global goal of Universal Health Coverage is a significant challenge since half of the world's population does not have access to essential health services (1). The Sustainable Development Goals

For peer review only - http://bmjopen.bmj.com/site/about/guidelines.xhtml

stresses the need for accelerating the ambitions to reach the targets for improving maternal, newborn, and child health (2). The World Health Organization, with its partners, has developed policies and programs aiming to promote health equity across and within nations (3). Utilization of maternal, newborn, and child health services has increased. However, inequities by geographic location and socio-economic group are challenges in the efforts towards universal health coverage (4) (5).

Studies from low-income African countries have indicated a strong association between geographic accessibility to health facilities and the use of maternal and child health services (6). In Ethiopia, reports have described geographical inequality. Distance measured as a straight line and walking time was associated with attendance to antenatal care and facility delivery (7–9). The topography of Ethiopia, characterized by difficult steep terrain with high hills, valleys and long distances from home to health facilities, has contributed to inequalities in the use of maternal and child health services (10). In addition, studies in Ethiopia and other African countries have shown that distance to health facilities was negatively associated with the use of family planning (11), antenatal care (12), facility deliveries (13), and care-seeking for sick children (14)

To the best of our knowledge, no previous studies have simultaneously analyzed the difference in altitude, the distance between household and health facility, as well as socio-economic characteristics and their possible association with utilization of a range of maternal and child health services in Ethiopia. Therefore, this study aimed to analyze if the geographic distance from home to the nearest health facility, the difference in altitude between home and health facility, and socio-economic status were associated with the use of antenatal care, facility delivery, full child immunization, and utilization of primary care services for sick children in rural Ethiopia.

For peer review only

For peer review only - http://bmjopen.bmj.com/site/about/guidelines.xhtml

METHODS

Study design and setting

We conducted a cross-sectional study in 46 districts of the four most populous Ethiopian regions, namely, Amhara, Oromia, Tigray, and Southern Nations, Nationalities, and Peoples (**Figure 1**). The regions have a population scattered in dispersed settlements, and the primary source of income is farming.

In the Ethiopian health delivery system, the primary health care unit is comprised of five satellite health posts and one health center. Each health post serves approximately 5,000 people and is staffed by two female Health Extension Workers.

The study was conducted from December 2018 to February 2019. The Ethiopian Public Health Institute, in collaboration with the London School of Hygiene and Tropical Medicine, University of Gondar, Mekelle University, Jimma University, and Hawassa University, conducted the survey.

Sampling

This study was using data from an evaluation of the Optimization of Health Extension Program intervention that was implemented in four regions of Ethiopia. This intervention aimed to improve care utilization for sick under-five children (15). The evaluation included baseline and endline surveys, and this study was a secondary analysis of end-line data.

A two-stage stratified cluster sampling was applied in the selected study districts. The first stage used lists of enumeration areas from the 2007 Ethiopian Housing and Population Census as the sampling frame (16). Then, 194 enumeration areas were selected with probability proportional to size.

In the second stage, all households within each enumeration area were listed, and a sampling interval was calculated. A random start number between one and the sampling interval was then selected. The households that matched the random start number on the list were chosen as the first household to be included. This process was repeated until the targeted number of 60 households in each cluster was reached. All women aged 13 to 49 years and children under the age of five years, who lived in the

For peer review only

selected households, were included in the study. The health posts and the health centers serving the enumeration area were included in the study.

We used a standard sample size formula to calculate the sample size. We estimated a design effect of 1.3, 80% power, and the assumption of a ratio of children less than five years of age per household was considered, based on sampling reported in the Ethiopia DHS 2011 (17). We used the assumption to detect 15% improved childcare utilization in two groups (intervention and comparison) between baseline and end-line surveys. The sample size was estimated to be 6,000 households per group (12,000 in total) to have 80% power to detect differences of 15 percentage points for care-seeking for 2-59 months children. The protocol for evaluation study was registered with a trial number ISRCTN12040912 and published (15).

Data collection tools

We used a modular questionnaire that was based on the Demographic and Health Survey and similar assessment tools. Information on the antenatal care attendance and place of delivery was collected from all women aged 13-49 who had a live birth in the last 12 months before the survey. Caregivers of children aged 2-59 months were asked whether the child had any illness in the previous two weeks, and if they had sought care from an appropriate provider. Caregivers were invited to show the immunization cards and asked additional questions on the different vaccinations if the card was not available. Besides, the questionnaire included information on socio-demographic data, assets, and geographic coordinates of households and health facilities. The coordinates were measured by GPS dongles (ND-100S).

Definition of variables

Outcome variables

We estimated the overall coverage of four selected maternal and child health services. For women, this included 1) the proportion of women aged 13 to 49 with a live birth within the last 12 months before the survey who had attended antenatal care four or more times, and 2) the proportion of women with a live

For peer review only - http://bmjopen.bmj.com/site/about/guidelines.xhtml

birth within 12 months before the survey who gave birth in a health facility. For the child health services utilization we included: 1) the proportion of children aged 12–23 months who had received full immunization, which was defined as Bacillus Calmette-Guerin (BCG), three pentavalent vaccinations (diphtheria, tetanus, pertussis, hepatitis B, and Haemophilus influenzae), oral polio vaccine, and one dose of measles vaccine (18), and 2) the proportion of children aged 2–59 months with fever, diarrhea, or suspected pneumonia (cough and difficult or fast breathing) in the last two weeks for whom care was sought from an appropriate provider, i.e., health posts, health centers, hospitals, or private clinics.

Explanatory variables

We used the Euclidian distance from the participants' houses to the nearest health facility, and household wealth as explanatory variables. Furthermore, we included other socio-demographic variables such as age, sex, parity, region, caregiver's education level, and number of children in the household to adjust for potential confounding in the analyses.

Data management and analysis

Household data on care utilization were linked with the GPS information of the 142 health centers posts and 164 health posts that served the households. The linking was done using cluster identification. The Euclidian distance from participants' houses to the nearest health facility was calculated using the geographical location of health facilities and households. Thus, we calculated the distance to the nearest health post for child health services (full child immunization and sick child health care utilization) and distance to the nearest health center for maternal health care services (antenatal care and facility delivery). These distances were divided into tertiles and labeled as short, medium, and long. Similarly, the difference in altitude was estimated using the altitude information of both the households and the health facility and classified as small, medium, and large. Corrections of the GPS readings were done whenever the reading erroneously was positioned outside the study areas. If that was the case, we adjusted by considering the nearest GPS reading captured. To estimate household wealth, we constructed wealth

For peer review only

For peer review only - http://bmjopen.bmj.com/site/about/guidelines.xhtml

index using 15 household assets and characteristics. We used principal component analysis (PCA) to construct the wealth index and divided it into tertiles (19).

We first analyzed the prevalence of the outcomes variables and graphically displayed the relation between distance, the difference in altitude, household wealth and facility visits for antennal care, facility delivery, child immunization, and sick child services utilization. After that, we used two-level mixed-effects logistic regression modeling to examine the associations with individual-level variables (wealth, number of children in the household, sex, age, parity, and educational status), group-level variables (region, distance, and the difference in altitude) and the interactions between the two. We analyzed the crude and adjusted odds ratios with 95% confidence intervals. The regression models accounted for confounding and clustering effects. The model quality was assessed using Akaki Information Criteria (AIC) and Intracluster Correlation Coefficient. The significance level was set at a P-value of less than 5%. All analyses were done using STATA 15 (Stata Corporation, College Station, Texas).

Patient and Public Involvement

Patients and the public were not involved in this study.

Ethics approval and consent to participate

We obtained ethical approval from the London School of Hygiene & Tropical Medicine, London, UK (Ethics Ref 16117), the Ethiopian Public Health Institute (Ethics Ref 613/52), and University of Gondar (O/V/P/RCS/05/214/2018). During the data collection, written informed consent was obtained from household heads and women, and assent from minors (age 13-18).

RESULTS

Characteristics of the study participants

Out of 11,877 women aged 13-49, 957 had given birth during 12 months before the survey. Of these, we linked 766 women (80%) with the geographic location data of the 142 nearest health centers, since antenatal and delivery services were provided at the health center level or above (**Table 1**). We collected information on 5,786 children from 2 to 59 months of age. Of these, we linked data on 5,285 (91%)

For peer review only - http://bmjopen.bmj.com/site/about/guidelines.xhtml

children with the geographic location of 164 nearest health posts. We estimated the Euclidian distance (median: 9.7, IQR:5.2-14.4 kilometer) and the difference in altitude (median: 54, IQR:24-1312 meter from the participants' houses to the nearest health facility.

Table 1: Number of women and children with data linked to the health facility Geographical Positioning System information that was used to calculate distance and difference in altitude between home and health facility

Group	Number of cases	Number of health facilities linked[1]	Cases linked[2]
Children 2-59 months	5787	164 health posts	5285 (91%)
Children 12-23 months	992	164 health posts	912 (92%)
Women 13-49 years	957	142 health centers	766 (80%)

[1]Number of health facilities assessed in the study area that were supposed to provide service to the study population
[2]Number of study participants whose data were linked to the health facility location to calculate the distance and difference in altitude between the household and the health facility

For peer review only

Utilization of maternal health services

Of the 766 mothers who had given birth in the previous 12 months before the survey, 39% (95% CI: 35-42) had attended antenatal care four or more times. The proportion of women who had delivered at a health facility was 55% (95% CI: 51-58). There was an inverse relationship between distance, the difference in attitude, and antenatal care or facility delivery (**Figure 2**). There was an increased proportion of prenatal visits and the percentage of facility deliveries with an increasing wealth index.

Utilization of child health services

Among the 912 children in the age group 12-23 months, 36% (95%CI: 33-39) were fully immunised. The two-week morbidity with common childhood illnesses, i.e., fever, diarrhea, or suspected pneumonia (cough with fast or difficult breathing), was assessed among 5,285 children from 2 to 59 months of age. Of these, 9.4% (95% CI: 8.6-10.2) were found to have been sick. The proportion of children who sought care from an appropriate provider, such as health post, health center, hospital, or private clinics, was 35% (95% CI: 31-39). There was no clear pattern in the associations between distance, the difference in altitude, wealth index, and child immunizations or utilization of services for sick children (**Figure 2**).

Determinants of maternal and child health services utilization

For peer review only - http://bmjopen.bmj.com/site/about/guidelines.xhtml

The multilevel regression analysis, after adjusting for age, parity, education, and region showed that women living with higher difference in altitude were less likely to have attended antenatal care four or more times (AOR=0.46; 95% CI: 0.29-0.74) and to have delivered at a health facility (AOR=0.33; 95% CI: 0.19-0.59), Table 2. Women living at a long distance from health facilities were less likely to have delivered at health facility (AOR=0.57; 95% CI: 0.34-0.96). Women from wealthier household were more likely to have received antenatal care four or more times (AOR=1.67; 95% CI: 1.02-2.75) and delivered at a health facility (AOR=3.70; 95% CI: 2.11-6.47) than their counterparts in the lower wealth tertiles.

Table 2: Determinants of antenatal care four or more times and facility delivery in four regions of Ethiopia, 2019.

Characteristic	Category	Antenatal care four or more visits		Institutional delivery	
		**OR (95% CI)**	**AOR (95% CI)**	**OR (95% CI)**	**AOR (95% CI)**
**Household and individual-level characteristics**					
Wealth tertiles	Low	Ref.	Ref.	Ref.	Ref.
	Middle	1.40(0.90,2.19)	1.15(0.74,1.81)	2.48(1.54,4.01)	1.61(0.99,2.60)
	Upper	2.50(1.55,4.02)	1.67(1.02,2.75)	6.85(3.87,12.15)	3.70(2.11,6.48)
Age	<20	Ref.	Ref.	Ref.	Ref.
	21-25	1.84(1.01,3.37)	1.52(0.80,2.89)	0.62(0.34,1.17)	0.72(0.36,1.43)
	26-30	2.33(1.29,4.22)	1.74(0.85,3.53)	0.71(0.38,1.32)	0.99(0.45,2.15)
	31-35	1.99(1.02,3.91)	1.51(0.68,3.40)	0.85(0.41,1.76)	1.35(0.54,3.34
	36-49	1.97(0.90,4.28)	1.42(0.56,3.61)	0.78(0.33,1.85)	1.34(0.47,3.82)
Educational level	No school	Ref.	Ref.	Ref.	Ref.
	Schooling	1.12(0.79,1.60)	1.18(0.80,1.74)	1.53(1.02-2.29)	1.26(0.81,1.96)
Parity	One	Ref.	Ref.	Ref.	Ref.
	Two	0.92(0.51,1.68)	1.01(0.55,1.87)	0.69(0.34,1.41)	0.94(0.46,1.93)
	Three	0.69(0.37,1.30)	0.77(0.39,1.52)	0.37(0.18,0.74)	0.48(0.22,1.05)
	Four	1.35(0.74,2.47)	1.44(0.73,2.84)	0.41(0.20,0.84)	0.62(0.28,1.40)
	≥Five	0.96(0.56,1.62)	1.01(0.56,2.15)	0.38(0.20,0.71)	0.47(0.21,1.03)
**Group-level characteristics**					
Region	Tigray	Ref.	Ref.	Ref.	Ref.
	Amhara	0.93(0.49,1.70)	0.75(0.41,1.36)	1.99(0.91,4.39)	1.35(0.66,2.77)
	Oromia	0.23(0.13,0.45)	0.22(0.12,0.41)	0.13(0.06,0.28)	0.13(0.06,0.26)
	SNNP	1.48(0.66,3.35)	1.05(0.49,3.34)	2.32(0.80,6.75)	1.26(0.48,3.34)
Distance to health facility tertiles	Short	Ref.	Ref.	Ref.	Ref.
	Medium	1.06(0.69,1.64)	1.01(0.67,1.54)	0.87(0.52,1.44)	0.90(0.56,1.47)
	Long	0.75(0.46,1.22)	0.77(0.49,1.19)	0.51(0.29,0.90)	0.57(0.34,0.96)
Difference in altitude tertiles[1]	Small	Ref.	Ref.	Ref.	Ref.
	Medium	0.78(0.49,1.22)	0.85(0.55,1.29)	0.59(0.34,1.02)	0.60(0.36,1.01)
	Great	0.48(0.28,0.83)	0.46(0.29,0.74)	0.39(0.19,0.79)	0.34(0.19,0.59)
ICC for the null model (%)			27%		49%
ICC for the final model (%)			8%		13%

[1]Difference in altitude between household and nearest health center

For peer review only - http://bmjopen.bmj.com/site/about/guidelines.xhtml

There was no clear pattern in the associations between distance from home to health post, the difference in altitude, or household wealth and child immunization and sick childcare utilization (Table 3). There was no indication of socio-economic inequity in coverage of immunization or utilization of services for the sick child. The medium distance stratum showed a lower coverage, but this could be a random error given the lack of consistency.

Table 3: Determinants of child immunization and sick child services utilization in four regions of Ethiopia; 2019.

Variable	Category	Full immunization 12-23 months		Sick child care utilization 2-59 months	
		OR (95% CI)	AOR (95% CI)	OR (95% CI)	AOR (95% CI)
**Household and individual-level characteristics**					
Wealth tertiles	Low	Ref.	Ref.	Ref.	Ref.
	Middle	0.91(0.52, 1.62)	0.75(0.42,1.34)	0.73(0.39,1.37)	0.71(0.38,1.33)
	Upper	1.58(0.91,2.77)	1.15(0.61,2.17)	1.02(0.55,1.91)	0.89(0.42,1.88)
Sex	Girl	Ref.	Ref.	Ref.	Ref.
	Boy	1.15(0.84,1.57)	1.18(0.85,1.63)	1.11(0.73,1.66)	1.02(0.67,1.55)
Number of under five children in the household	One child	Ref.	Ref.	Ref.	Ref.
	≥Two children	1.10(.79, 1.54)	1.21(.86,1.72)	1.15(0.74,1.80)	1.33(0.83,2.14)
**Group-level characteristics**					
Region	Tigray	Ref.	Ref.	Ref.	Ref.
	Amhara	1.32(0.72,2.42)	1.23(0.62,2.45)	2.07(1.03,4.13)	2.26(1.02,5.03)
	Oromia	0.49(0.25,0.94)	0.49(0.25,0.95)	2.87(1.42,5.86)	3.09(1.54,6.19)
	SNNP	0.68(0.30,1.52)	0.55(0.23,1.34)	2.04(0.90,4.62)	2.55(1.07,6.09)
Distance to health facility tertiles	Short	Ref.	Ref.	Ref.	Ref.
	Medium	0.69(0.46,1.04)	0.64(0.41,0.98)	0.49(0.29,0.82)	0.50(0.29,0.85)
	Long	0.84(0.55,1.28)	0.82(0.53,1.27)	0.76(0.44,1.29)	0.76(0.44,1.29)
Difference in altitude tertiles[1]	Small	Ref.	Ref.	Ref.	Ref.
	Medium	1.00(0.66,1.47)	0.99(0.65,1.51)	0.88(0.54,1.44)	1.02(0.61,1.70)
	Great	0.85(0.54,1.35)	0.89(0.55,1.42)	0.48(0.28,0.82)	0.59(0.34,1.01)
ICC for the null model (%)			24%		18%
ICC for the final model (%)			19%		8%

[1]Difference in altitude between household and nearest health facility

DISCUSSION

We have shown that long distance to the health center, a more significant difference in altitude between home and facility, and a lower socio-economic group were all associated with a smaller proportion of women delivering at a health facility. Similarly, those living with a considerable difference in altitude and belonging to a lower socio-economic group were less likely to have attended antenatal care four times or

For peer review only - http://bmjopen.bmj.com/site/about/guidelines.xhtml

For peer review only

more. However, distance, altitude, or socio-economic status were neither associated with coverage of child immunization nor with care-seeking for sick children.

An estimated 39% of the pregnant women in the current study had attended antenatal care four or more times. This proportion was higher than the corresponding result reported from the same study areas two years earlier, which showed coverage at 30% (10). It was a bit lower than the national average, based on the Demographic and Health Survey 2019, which was 43% (20). This increase could reflect the government's efforts towards universal health coverage (21). In contrast to the current study, in an analysis of the 2016 Ethiopian Demographic and Health Survey, distance to the health center was associated with four or more antenatal care visits (7). Furthermore, in an analysis of data from Demographic and Health Surveys in five East African counties 2010-2014 showed that geographical accessibility of health facilities had a strong influence on maternal health care utilization (22). The study employed cost-distance analysis based on the health facilities' position and difficulties in traversing the surface of each 300x300 m cell in the study areas. The analysis included the slopes based on elevation measurements as well as the distance from home to the health facility. These findings imply that efforts climbing up and down the hills were barriers to accessing these services. Similar result was shown regarding the influence of household wealth on antenatal care use and facility delivery in the previous study in the same study area (19).

We found that more than half of the study women had delivered at a health facility, which was similar to the coverage (48%) reported by the 2019 Ethiopian Demographic Health Survey (20). In line with the current study, there was also a marked wealth-based inequity in the utilization of antenatal care four or more times and facility delivery in a study performed in the same areas two years earlier (19). Reviews of similar Ethiopian studies (23), research from sub-Saharan Africa countries (24) and East African countries (22), as well as from a range of low-income countries (25) point at considerable regional variation, social inequities in the utilization of facility delivery services, and the importance of topography

and distance from home to health centers and hospitals. A study from Kenya which attempted to disentangle distance to facility and quality of care indicated that pregnant women bypassed possible places to deliver, preferring to give birth at a facility that provided better-quality care (26). Furthermore, an analysis from 81 low and middle-income countries suggested that closing the quality gap would enhance the use of services (27).

In terms of utilization of child health services, thirty-six percent of children aged 12-23 months had received all basic immunizations. This low level was similar to the national estimate (38%) from 2019 Ethiopian mini-Demographic and Health Survey (18). A study two years earlier in the same rural areas as the current study showed an equitable distribution of full child immunization across different socio-economic groups (19). This relative equity in vaccination coverage could be explained by the rural outreach services, organized from the health posts, where eligible children were summoned to vaccination days in their local villages (19). A study in Ethiopia's hard-to-reach areas, distance to the health facility influenced full child immunization coverage in addition to the mother's education, utilization antenatal delivery services and child delivered at the health facility (28). However, in an analysis of the 2016 Demographic and Health Survey, full child immunization showed significant variation between groups characterized by geographic, socioeconomic, and maternal factors with pro-advantaged bias (29). The immunizations are given at the health post and partly through outreach services in the villages. This combination of facility-based and outreach services may theoretically not only increase coverage but also enhance equity (30,31).

One-third of the children 2-59 months, who had been sick in the two weeks before the survey, had sought care from health posts, health centers, hospitals or private clinics which is in line with the national estimates of the utilization of health services for sick children (32). Our results showed no association between care-seeking for sick children and distance to the nearest health center, difference in altitude and socio-economic status. During the last decade, Ethiopia has scaled up the integrated Community Case

For peer review only - http://bmjopen.bmj.com/site/about/guidelines.xhtml

Management of common childhood illnesses provided by salaried health extension workers at the health posts (33). The availability of community-level primary care services at health posts close to home, offering child health services, could partly explain the relative equity in utilization. Contrary to this, the 2016 nationally representative Demographic Health Survey showed inequities in care-seeking for common childhood illnesses between groups defined by geographic region, mother's education, or household wealth (32). The Ethiopian government has expanded maternal, neonatal, and child health services in the country (34,35). Further efforts are needed not only to expand these services but also to increase the quality of care (36,37) to reach the goal of Universal Health Coverage (21).

CONCLUSION

The topography, i.e., the distance and difference in altitude of the areas, and socio-economic status of the household affected the utilization of maternal health services. We did not find such inequities in the utilization of child health services, although coverage was on a low level. The strive for universal health coverage in Ethiopia will require not only increased coverage but also targeted efforts to address the geographic and social differentials in care utilization.

For peer review only

List of abbreviations

AOR	Adjusted Odds Ratio
GPS	Geographical Positioning System
ICC	Intra-class Correlation Coefficient

Declaration

Funding

The study was funded by a grant to London School of Hygiene & Tropical Medicine from the Bill and Melinda Gates Foundation (OPP1132551)

Availability of data

Data could be available at a reasonable request to the corresponding author, Atkure Defar, atkuredefar@gmail.com

Authors' contributions

AD, LÅP and KA developed the idea of this analysis. AD analyzed and drafted the paper. All authors contributed to the interpretation of results, revision of the manuscript, and approval of the final version of the paper.

Consent for publication

Not applicable

Competing interests

The authors declare that they have no competing interests.

Acknowledgements

The authors would like to thank the University of Gondar for the support provided to the project. We are also grateful to the regional health bureaus and district health offices in the study areas for their smooth and kind cooperation. Special thanks to all field staff for their excellent fieldwork and to the study participants for their kind cooperation in interviews and data collection.

For peer review only

REFERENCES

1. World Health Organization, The World Bank. Tracking Universal Health Coverage - First Global Monitoring Report. Geneva, WHO; 2015.

2. Boerma T, Requejo J, Victora CG, Amouzou A, George A, Agyepong I, et al. Countdown to 2030: tracking progress towards universal coverage for reproductive, maternal, newborn, and child health. Lancet. 2018 Apr 14;391(10129):1538–48. https://doi.org/10.1016/S0140-6736(18)30104-1

3. Schaaf H, Hesse G. Schwindelerkrankungen im kindesalter: Erweiterte diagnosemöglichkeiten der vestibulären komponente. Vol. 79, Padiatrische Praxis. 2012. p. 253–65.

4. Memirie ST, Verguet S, Norheim OF, Levin C, Johansson KA. Inequalities in utilization of maternal and child health services in Ethiopia: The role of primary health care Health systems and services in low and middle income settings. BMC Health Serv Res. 2016;16(1):1–8. http://dx.doi.org/10.1186/s12913-016-1296-7

5. Kassile T, Lokina R, Mujinja P, Mmbando BP. Determinants of delay in care seeking among children under five with fever in Dodoma region, central Tanzania: a cross-sectional study. Malar J. 2014 Sep;13:348.

6. Lohela TJ, Campbell OMR, Gabrysch S. Distance to Care, Facility Delivery and Early Neonatal Mortality in Malawi and Zambia. PLoS One. 2012;7(12).

7. Tegegne TK, Chojenta C, Getachew T, Smith R, Loxton D. Antenatal care use in Ethiopia: A spatial and multilevel analysis. BMC Pregnancy Childbirth. 2019;19(1):399.

8. Tadele N, Lamaro T. Utilization of institutional delivery service and associated factors in Bench Maji zone, Southwest Ethiopia: community based, cross sectional study. BMC Health Serv Res. 2017;17(1):1–10.

9. Jimma R, District H, Zegeye K, Gebeyehu A, Melese T. Primary Health Care : Open Access The Role of Geographical Access in the Utilization of Institutional Delivery. Prim Heal care. 2014;4(1):1–6.

10. Defar A, Okwaraji YB, Tigabu Z, Persson LÅ, Alemu K. Geographic differences in maternal and child health care utilization in four Ethiopian regions; a cross-sectional study. Int J Equity Health. 2019;18(1):173.

11. Shiferaw S, Spigt M, Seme A, Amogne A, Skrøvseth S, Desta S, et al. Does proximity of women to facilities with better choice of contraceptives affect their contraceptive utilization in rural Ethiopia? PLoS One. 2017;12(11).

12. Tanou M, Kamiya Y. Assessing the impact of geographical access to health facilities on maternal healthcare utilization: Evidence from the Burkina Faso demographic and health survey 2010. BMC Public Health. 2019;19(1):1–8.

13. Nesbitt RC, Lohela TJ, Soremekun S, Vesel L, Manu A, Okyere E, et al. The influence of distance and quality of care on place of delivery in rural Ghana. Sci Rep. 2016;6(August):1–8. http://dx.doi.org/10.1038/srep30291

14. Sarrassat S, Meda N, Badolo H, Ouedraogo M, Some H, Cousens S. Distance to care, care seeking and child mortality in rural Burkina Faso: findings from a population-based cross-sectional survey. Trop Med Int Health. 2019 Jan;24(1):31–42.

For peer review only - http://bmjopen.bmj.com/site/about/guidelines.xhtml

15. Berhanu D, Okwaraji YB, Belayneh AB, Lemango ET, Agonafer N, Birhanu BG et al. Protocol for the evaluation of a complex intervention aiming at increased utilization of primary child health services in Ethiopia. A before and after study in intervention and comparison areas. BMC Health Serv Res. 2020;20(1):339–12.

16. Central Statistical Agency (CSA). Population Census Commission II [Internet]. Mortality. 2007. Available from: http://www.csa.gov.et/surveys/Population and Housing census/ETH-pop-2007/survey0/data/Doc/Reports/National_Statistical.pdf

17. Central Statistical Agency (CSA) and ICF International. Ethiopia Demographic and Health Survey, 2011. Addis Ababa, Ethiopia; 2011.

18. Tamirat KS, Sisay MM. Full immunization coverage and its associated factors among children aged 12-23 months in Ethiopia: Further analysis from the 2016 Ethiopia demographic and health survey. BMC Public Health. 2019;19(1):1–7.

19. Wuneh AD, Medhanyie AA, Bezabih AM, Persson LÅ, Schellenberg J, Okwaraji YB. Wealth-based equity in maternal, neonatal, and child health services utilization: A cross-sectional study from Ethiopia. Int J Equity Health. 2019;18(1):1–9.

20. Ethiopian Public Health Institute (EPHI) and ICF. 2019. Mini Demographic and Health Survey 2019: key Indicators. Rockville, Maryland, USA: EPHI and ICF. 2019.

21. Alebachew A, Hatt L, Kukla M. Monitoring and Evaluating Progress towards Universal Health Coverage in Ethiopia. PLoS Med. 2014;11(9):9–11.

22. Ruktanonchai CW, Ruktanonchai NW, Nove A, Lopes S, Pezzulo C, Bosco C, et al. Equality in maternal and newborn health: Modelling geographic disparities in utilisation of care in five East African countries. PLoS One. 2016;11(8):1–17.

23. Kebede A, Hassen K, Teklehaymanot AN. Factors associated with institutional delivery service utilization in Ethiopia. Int J Womens Health. 2016;8:463–75.

24. Moyer CA, Mustafa A. Drivers and deterrents of facility delivery in sub-Saharan Africa: A systematic review. Reprod Health. 2013;10(1):40.

25. Karra M, Fink G, Canning D. Facility distance and child mortality: a multi-country study of health facility access, service utilization, and child health outcomes. Int J Epidemiol. 2017 Jun;46(3):817–26.

26. Escamilla V, Calhoun L, Winston J, Speizer IS. The Role of Distance and Quality on Facility Selection for Maternal and Child Health Services in Urban Kenya. J Urban Health. 2018 Feb;95(1):1–12.

27. Chou VB, Walker N, Kanyangarara M. Estimating the global impact of poor quality of care on maternal and neonatal outcomes in 81 low- And middle-income countries: A modeling study. PLoS Med. 2019;16(12):1–16.

28. Girmay A, Dadi AF. Full Immunization Coverage and Associated Factors among Children Aged 12-23 Months in a Hard-to-Reach Areas of Ethiopia. Int J Pediatr. 2019;2019:1–8.

29. Geweniger A, Abbas KM. Childhood vaccination coverage and equity impact in Ethiopia by socioeconomic, geographic, maternal, and child characteristics. Vaccine. 2020;38(20):3627–38.

For peer review only

https://doi.org/10.1016/j.vaccine.2020.03.040

30. Oyo-Ita A, Cs W, Oringanje C, Ce N, Oduwole O, Mm M. Interventions for improving coverage of childhood immunisation in low- andmiddle-income countries. Cochrane Database Syst Rev. 2016;(7). https://www.ncbi.nlm.nih.gov/pubmed/27394698

31. Utazi CE, Thorley J, Alegana VA, Ferrari MJ, Takahashi S, Metcalf CJE, et al. Mapping vaccination coverage to explore the effects of delivery mechanisms and inform vaccination strategies. Nat Commun. 2019;10(1):1–10. http://dx.doi.org/10.1038/s41467-019-09611-1

32. ICF ACS (CSA) [Ethiopis]. Ethiopia Demographic and Health Survey 2016. 2016. Addis Ababa, Ethiopia, and Rockville, Maryland, US.

33. Miller NP, Amouzou A, Tafesse M, Hazel E, Legesse H, Degefie T, et al. Integrated Community Case Management of Childhood Illness in Ethiopia : Implementation Strength and Quality of Care. Am J Trop Med Hyg. 2014;91(2):424–34.

34. Byrne A, Hodge A, Jimenez-Soto E, Morgan A. What works? Strategies to increase reproductive, maternal and child health in difficult to access mountainous locations: A systematic literature review. PLoS One. 2014;9(2).

35. Karim AM, Tamire A, Medhanyie AA, Betemariam W. Changes in equity of maternal, newborn, and child health care practices in 115 districts of rural Ethiopia: Implications for the health extension program. BMC Pregnancy Childbirth. 2015;15(1):1–11. http://dx.doi.org/10.1186/s12884-015-0668-z

36. Allen E, Schellenberg J, Berhanu D, Cousens S, Marchant T. Associations between increased intervention coverage for mothers and newborns and the number and quality of contacts between families and health workers: An analysis of cluster level repeat cross sectional survey data in Ethiopia. PLoS One. 2018;13(8):1–13.

37. Defar A, Getachew T, Taye G, Tadele T, Getnet M, Shumet T, et al. Quality antenatal care services delivery at health facilities of Ethiopia, assessment of the structure/input of care setting. BMC Health Serv Res. 2020;20(1):485.

38. Ayalneh AA, Fetene DM, Lee TJ. Inequalities in health care utilization for common childhood illnesses in Ethiopia: evidence from the 2011 Ethiopian Demographic and Health Survey. Int J Equity Health. 2017;16(1):67. http://equityhealthj.biomedcentral.com/articles/10.1186/s12939-017-0561-7

39. Alene M, Yismaw L, Berelie Y, Kassie B. Health care utilization for common childhood illnesses in rural parts of Ethiopia: Evidence from the 2016 Ethiopian demographic and health survey. BMC Public Health. 2019;19(1):1–12.

For peer review only

Figure legend

Figure 1 Maps showing A) Ethiopia, B) the four study regions, and C) the 46 study districts in the four regions (Graph constructed in ArcGIS 10.4 software using freely available data from the Ethiopian Central Statistics Agency)

Figure 2: The distribution of antenatal care, facility delivery, child immunization and sick childcare utilization by tertiles of the distance and difference in altitude between the household and the nearest health facility, and the wealth index.

For peer review only

For peer review only - http://bmjopen.bmj.com/site/about/guidelines.xhtml

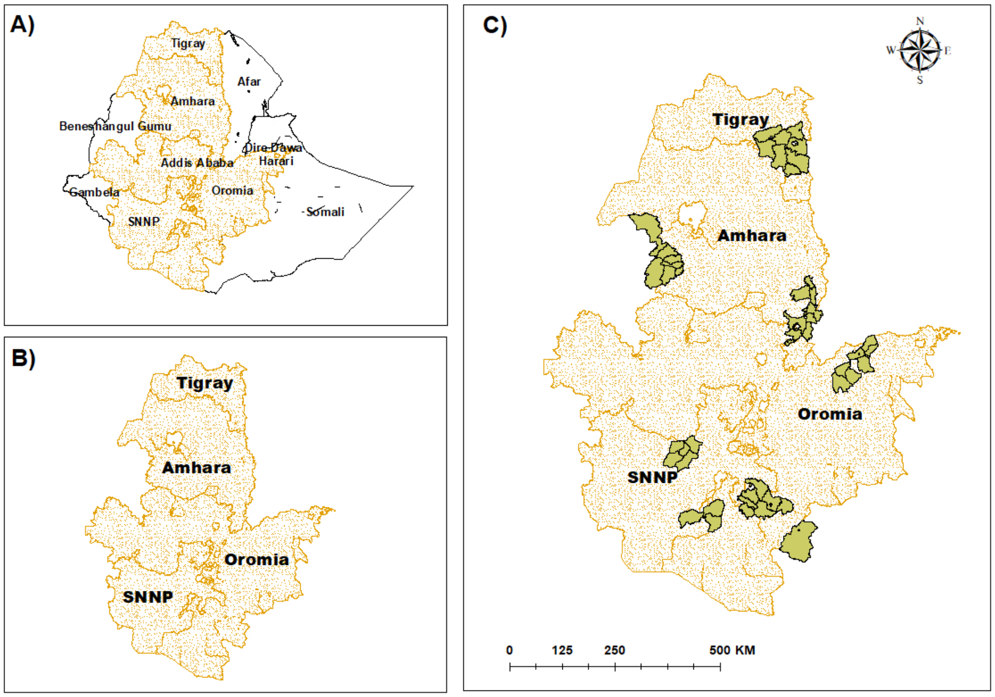

296x210mm (96 x 96 DPI)

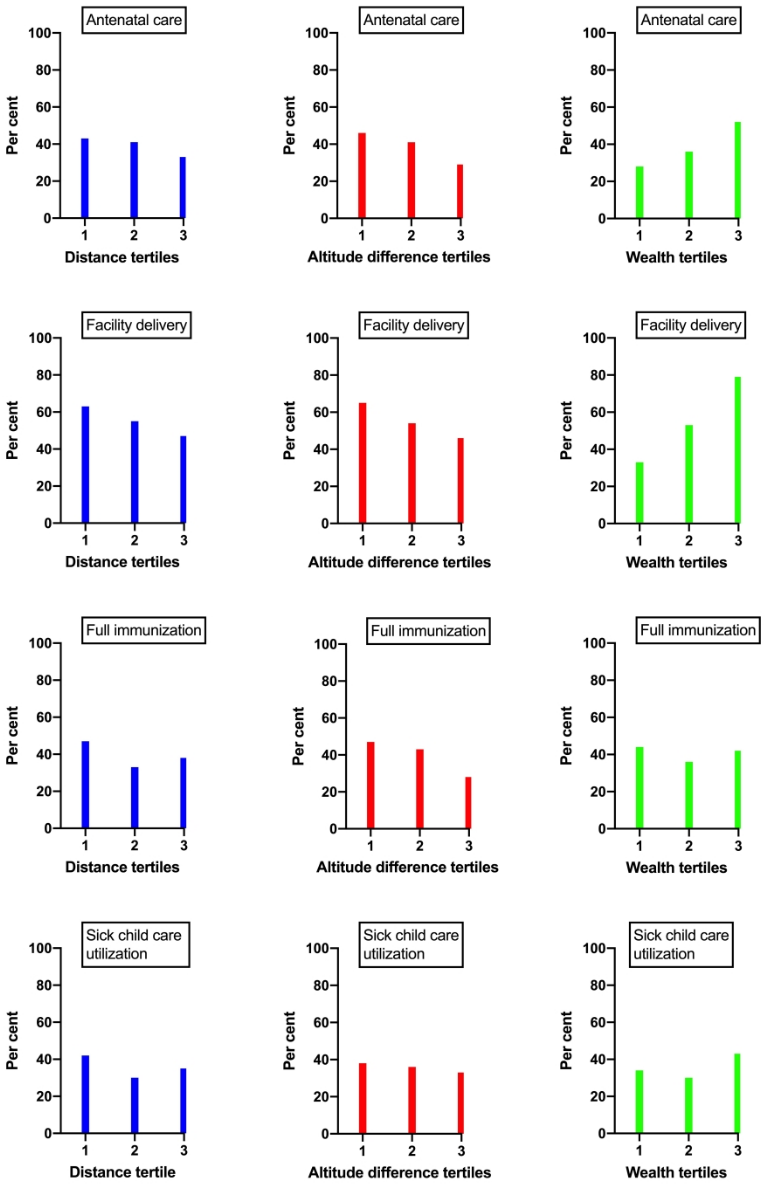

178x269mm (600 x 600 DPI)

STROBE—Checklist of items for a cross-sectional studies

**Manuscript section**	**Item No**	**Description**	**Reported** ✓	**Page #**
**Title and abstract**	1	(*a*) Indicate the study's design with a commonly used term in the title or the abstract		
		(*b*) Provide in the abstract an informative and balanced summary of what was done and what was found	✓	2
**Introduction**				
Background/rationale	2	Explain the scientific background and rationale for the investigation being reported	✓	4-5
Objectives	3	State specific objectives, including any prespecified hypotheses	✓	5
**Methods**				
Study design	4	Present key elements of study design early in the paper	✓	6
Setting	5	Describe the setting, locations, and relevant dates, including periods of recruitment, exposure, follow-up, and data collection	✓	6
Participants	6	(*a*) Give the eligibility criteria, and the sources and methods of selection of participants	✓	6-7
Variables	7	Clearly define all outcomes, exposures, predictors, potential confounders, and effect modifiers. Give diagnostic criteria, if applicable	✓	7
Data sources/ measurement	8*	For each variable of interest, give sources of data and details of methods of assessment (measurement). Describe comparability of assessment methods if there is more than one group	✓	*2 & 7*
Bias	9	Describe any efforts to address potential sources of bias	✓	4
Study size	10	Explain how the study size was arrived at		
Quantitative variables	11	Explain how quantitative variables were handled in the analyses. If applicable, describe which groupings were chosen and why	✓	6-7
Statistical methods	12	(*a*) Describe all statistical methods, including those used to control for confounding	✓	8
		(*b*) Describe any methods used to examine subgroups and interactions		
		(*c*) Explain how missing data were addressed	✓	8
		(*d*) If applicable, describe analytical methods taking account of sampling strategy	✓	8
		(*e*) Describe any sensitivity analyses		
**Results**				
Participants	13*	(a) Report numbers of individuals at each stage of study—eg numbers potentially eligible, examined for eligibility, confirmed eligible, included in the study, completing follow-up, and analysed	✓	9-10
		(b) Give reasons for non-participation at each stage		

For peer review only

		(c) Consider use of a flow diagram		
Descriptive data	14*	(a) Give characteristics of study participants (eg demographic, clinical, social) and information on exposures and potential confounders	✓	10
		(b) Indicate number of participants with missing data for each variable of interest	✓	10
Outcome data	15*	Report numbers of outcome events or summary measures	✓	10
Main results	16	(*a*) Give unadjusted estimates and, if applicable, confounder-adjusted estimates and their precision (eg, 95% confidence interval). Make clear which confounders were adjusted for and why they were included	✓	11
		(*b*) Report category boundaries when continuous variables were categorized	✓	10
		(*c*) If relevant, consider translating estimates of relative risk into absolute risk for a meaningful time period		
Other analyses	17	Report other analyses done—eg analyses of subgroups and interactions, and sensitivity analyses		
**Discussion**				
Key results	18	Summarise key results with reference to study objectives	✓	12-13
Limitations	19	Discuss limitations of the study, taking into account sources of potential bias or imprecision. Discuss both direction and magnitude of any potential bias	✓	4
Interpretation	20	Give a cautious overall interpretation of results considering objectives, limitations, multiplicity of analyses, results from similar studies, and other relevant evidence	✓	12-14
Generalisability	21	Discuss the generalisability (external validity) of the study results	✓	14
**Other information**				
Funding	22	Give the source of funding and the role of the funders for the present study and, if applicable, for the original study on which the present article is based	✓	16

*Give information separately for exposed and unexposed groups.

Note: An Explanation and Elaboration article discusses each checklist item and gives methodological background and published examples of transparent reporting. The STROBE checklist is best used in conjunction with this article (freely available on the Web sites of PLoS Medicine at http://www.plosmedicine.org/, Annals of Internal Medicine at http://www.annals.org/, and Epidemiology at http://www.epidem.com/). Information on the STROBE Initiative is available at www.strobe-statement.org.

For peer review only

BMJ Open

Distance, difference in altitude, and socio-economic determinants of utilization of maternal and child health services in Ethiopia: a geographic and multilevel modelling analysis

Journal:	*BMJ Open*
Manuscript ID	bmjopen-2020-042095.R1
Article Type:	Original research
Date Submitted by the Author:	12-Nov-2020
Complete List of Authors:	Defar, Atkure ; Ethiopian Public Health Institute; University of Gondar College of Medicine and Health Sciences, Institute of Public Health, Department of Epidemiology and Biostatistics<br>Okwaraji, Yemisrach; London School of Hygiene and Tropical Medicine Faculty of Infectious and Tropical Diseases, Department of Disease Control; Ethiopian Public Health Institute<br>Tigabu, Zemene; University of Gondar College of Medicine and Health Sciences, Department of Paediatrics and Child Health<br>Persson, Lars; London School of Hygiene and Tropical Medicine Faculty of Infectious and Tropical Diseases, Department of Disease Control; Ethiopian Public Health Institute<br>Alemu, Kassahun ; University of Gondar College of Medicine and Health Sciences, Institute of Public Health, Department of Epidemiology and Biostatistics
**Primary Subject Heading**:	Public health
Secondary Subject Heading:	Health services research, Public health, Epidemiology
Keywords:	HEALTH SERVICES ADMINISTRATION & MANAGEMENT, Community child health < PAEDIATRICS, PUBLIC HEALTH

SCHOLARONE™
Manuscripts

I, the Submitting Author has the right to grant and does grant on behalf of all authors of the Work (as defined in the below author licence), an exclusive licence and/or a non-exclusive licence for contributions from authors who are: i) UK Crown employees; ii) where BMJ has agreed a CC-BY licence shall apply, and/or iii) in accordance with the terms applicable for US Federal Government officers or employees acting as part of their official duties; on a worldwide, perpetual, irrevocable, royalty-free basis to BMJ Publishing Group Ltd ("BMJ") its licensees and where the relevant Journal is co-owned by BMJ to the co-owners of the Journal, to publish the Work in this journal and any other BMJ products and to exploit all rights, as set out in our licence.

The Submitting Author accepts and understands that any supply made under these terms is made by BMJ to the Submitting Author unless you are acting as an employee on behalf of your employer or a postgraduate student of an affiliated institution which is paying any applicable article publishing charge ("APC") for Open Access articles. Where the Submitting Author wishes to make the Work available on an Open Access basis (and intends to pay the relevant APC), the terms of reuse of such Open Access shall be governed by a Creative Commons licence – details of these licences and which Creative Commons licence will apply to this Work are set out in our licence referred to above.

Other than as permitted in any relevant BMJ Author's Self Archiving Policies, I confirm this Work has not been accepted for publication elsewhere, is not being considered for publication elsewhere and does not duplicate material already published. I confirm all authors consent to publication of this Work and authorise the granting of this licence.

For peer review only

Distance, difference in altitude, and socio-economic determinants of utilization of maternal and child health services in Ethiopia: a geographic and multilevel modelling analysis

Atkure Defar[1, 4*], Yemisrach B. Okwaraji[1, 3], Zemene Tigabu[2], Lars Åke Persson[1, 3], Kassahun Alemu[4]

*Corresponding author:

Atkure Defar, MPH, MBA, Email: atkuredefar@gmail.com
ORCID, https://orcid.org/0000-0001-9435-2135

Co-authors:

Yemisrach Okwaraji, PhD, Email, yemisrach.okwaraji@lshtm.ac.uk
ORCID, https://orcid.org/0000-0002-4541-2856
Zemene Tigabu MD/Paediatrician, Email: zemene.tigabu@gmail.com
ORCID, https://orcid.org/0000-0001-6040-032X
Prof. Lars Åke Persson; MD, PhD, Email: Lars.Persson@lshtm.ac.uk
ORCID, https://orcid.org/0000-0003-0710-7954
Kassahun Alemu Gelaye, PhD, Email: kassalemu@gmail.com
ORCID, https://orcid.org/0000-0002-8360-0292

Affiliation

[1]Ethiopian Public Health Institute, Addis Ababa, Ethiopia

[2]Department of Paediatrics and Child Health, School of Medicine, College of Medicine and Health Sciences, University of Gondar, Gondar, Ethiopia

[3]London School of Hygiene and Tropical Medicine, London, UK

[4]Department of Epidemiology and Biostatistics, Institute of Public Health, College of Medicine and Health Sciences, University of Gondar, Gondar, Ethiopia

Word count 3372

For peer review only

ABSTRACT

Objective We assessed whether geographic distance and difference in altitude between home to health facility and household socio-economic status were associated with utilization of maternal and child health services in rural Ethiopia.

Design Household and health facility surveys were conducted from December 2018 to February 2019.

Setting Fortysix districts in the Ethiopian regions Amhara, Oromia, Tigray, and Southern Nations, Nationalities, and Peoples.

Participants A total of 11,877 women aged 13-49 years and 5,786 children aged 2-59 months were included.

Outcome measures The outcomes were four or more antenatal care visits, facility delivery, full child immunization, and utilization of health services for sick children. A multilevel analysis was carried out with adjustments for potential confounding factors.

Results Overall, 39% (95% CI: 35-42) women had attended four or more antenatal care visits, and 55% (95%CI: 51-58) delivered at health facilities. One in three (36%, 95%CI: 33-39) of children had received full-immunizations and 35% (95% CI: 31-39) of sick children used health services. A long distance (AOR=0.57; 95% CI 0.34-0.96), and larger difference in altitude (AOR=0.34; 95%CI 0.19-0.59) were associated with fewer facility deliveries. Larger difference in altitude was associated with a lower proportion of antenatal care visits (AOR=0.46; 95%CI: 0.29-0.74). A higher wealth index was associated with a higher proportion of antenatal care visits (AOR=1.67; 95%CI: 1.02-2.75) and health facility deliveries (AOR=2.11; 95%CI: 2.11-6.48). There was no association between distance, difference in altitude, or wealth index and children being fully immunized or seeking care when they were sick.

Conclusion Achieving universal access to maternal and child health services will require not only strategies to increase coverage but also targeted efforts to address the geographic and socio-economic differentials in care utilization, especially for maternal health.

For peer review only

Keywords: Distance, Altitude, Antenatal care, Facility delivery, Immunization coverage, Sick child services utilization, Ethiopia

Article summary

Strength and limitation of this study

- We assessed geographic and social equity across a continuum of maternal and child healthcare, i.e., antenatal care visits, facility delivery, immunization, and utilization of health services for sick children.
- We used a multi-level regression modelling that included the determinants, the outcome, and potential confounding factors at the cluster, household, and individual levels that accounted for confounding and clustering effects.
- To reduce the risk of recall bias, reporting of utilisation of maternal services were limited to the last 12 months and care-seeking for common childhood illnesses to a two-week recall period.
- To make our results comparable with other studies, such as the Demographic and Health Surveys, reporting of immunization status was limited to children aged 12-23 months.
- In addition to the straight-line distance between home and the nearest health facility, we assessed the difference in altitude between home and the nearest health facility as a potentially important dimension of distance that could affect care utilization.

INTRODUCTION

The global goal of Universal Health Coverage is a significant challenge since half of the world's population does not have access to essential health services (1). The Sustainable Development Goals stresses the need for accelerating the ambitions to reach the targets for improving maternal, newborn, and child health (2). The World Health Organization, with its partners, has developed policies and programs aiming to promote health equity across and within nations (3). Utilization of maternal, newborn, and child

For peer review only - http://bmjopen.bmj.com/site/about/guidelines.xhtml

health services has increased. However, inequities by geographic location and socioeconomic status are challenges in the efforts towards universal health coverage (4,5).

Studies from low-income African countries have indicated a strong association between geographic accessibility to health facilities and the use of maternal and child health services (6). In Ethiopia, reports have described geographical inequality. Distance measured as a straight line and walking time was associated with attendance to antenatal care and facility delivery (7–9). The topography of Ethiopia, characterized by difficult steep terrain with high hills, valleys and long distances from home to health facilities, has contributed to inequalities in the use of maternal and child health services (10). In addition, studies in Ethiopia and other African countries have shown that distance to health facilities was negatively associated with the use of family planning (11), antenatal care (12), facility deliveries (13), and care-seeking for sick children (14)

To the best of our knowledge, no previous studies have simultaneously analysed the difference in altitude, the distance between household and health facility, as well as socio-economic characteristics and their possible association with utilization of a range of maternal and child health services in Ethiopia. Therefore, this study aimed to analyse if the geographic distance from home to the nearest health facility, the difference in altitude between home and health facility, and socio-economic status were associated with the use of antenatal care, facility delivery, full child immunization, and utilization of primary care services for sick children in rural Ethiopia.

METHODS

Study design and setting

We conducted a cross-sectional study in 46 districts of the four most populous Ethiopian regions, namely, Amhara, Oromia, Tigray, and Southern Nations, Nationalities, and Peoples (**Figure 1**). The regions have a population scattered in dispersed settlements, and the primary source of income is farming.

For peer review only - http://bmjopen.bmj.com/site/about/guidelines.xhtml

For peer review only

In the Ethiopian health delivery system, the primary health care unit is comprised of five satellite health posts and one health centre. Each health post serves approximately 5,000 people and is staffed by two female Health Extension Workers.

The study was conducted from December 2018 to February 2019. The Ethiopian Public Health Institute, in collaboration with the London School of Hygiene and Tropical Medicine, University of Gondar, Mekelle University, Jimma University, and Hawassa University, conducted the survey.

Sampling

This study used data from an evaluation of the Optimization of Health Extension Program intervention that was implemented in four regions of Ethiopia. This intervention aimed to improve care utilization for sick under-five children (15). The evaluation included baseline and endline surveys, and this study was a secondary analysis of end-line data.

A two-stage stratified cluster sampling was applied in the selected study districts. The first stage used lists of enumeration areas from the 2007 Ethiopian Housing and Population Census as the sampling frame (16). Thereafter, 194 enumeration areas were selected with probability proportional to size (sampling from a finite population in which a size measure is available for each population unit before sampling and where the probability of selecting a unit is proportional to its size.

In the second stage, all households within each enumeration area were listed, and a sampling interval was calculated. A random start number between one and the sampling interval was selected. The households that matched the random start number on the list were chosen as the first household to be included. This process was repeated until the targeted number of 60 households in each cluster was reached. All women aged 13 to 49 years and children under the age of five years, who lived in the selected households, were included in the study. The health posts and the health centres serving the enumeration area were included in the study.

For peer review only - http://bmjopen.bmj.com/site/about/guidelines.xhtml

We used a standard sample size formula to calculate the sample size. We estimated a design effect of 1.3, 80% power, and the assumption of a ratio of children less than five years of age per household was considered, based on sampling reported in the Ethiopia DHS 2011 (17). We used the assumption to detect 15% improved childcare utilization in two groups (intervention and comparison) between baseline and end-line surveys. The sample size was estimated to be 6,000 households per group (12,000 in total) to have 80% power to detect differences of 15 percentage points for care-seeking for 2-59 months children. The protocol for the evaluation study was registered with a trial number ISRCTN12040912 and published (15).

Data collection tools

We used a modular questionnaire that was based on the Demographic and Health Survey and similar assessment tools. Information on the antenatal care attendance and place of delivery was collected from all women aged 13-49 who had a live birth in the last 12 months before the survey. Caregivers of children aged 2-59 months were asked whether the child had any illness in the previous two weeks and if they had sought care from an appropriate provider. Caregivers were invited to show the immunization cards and asked additional questions on the different vaccinations if the card was not available. Besides, the questionnaire included information on socio-demographic data, assets, and geographic coordinates of households and health facilities. The coordinates were measured by GPS dongles (ND-100S).

Definition of variables

Outcome variables

We estimated the overall coverage of four selected maternal and child health services. For women, this included 1) the proportion of women aged 13 to 49 with a live birth within the last 12 months before the survey who had attended antenatal care four or more times, and 2) the proportion of women with a live birth within 12 months before the survey who gave birth in a health facility. For the child health services utilization we included: 1) the proportion of children aged 12–23 months who had received full

For peer review only

immunization, which was defined as Bacillus Calmette-Guerin (BCG), three pentavalent vaccinations (diphtheria, tetanus, pertussis, hepatitis B, and Haemophilus influenzae), oral polio vaccine, and one dose of measles vaccine (18), and 2) the proportion of children aged 2–59 months with fever, diarrhoea, or suspected pneumonia (cough and difficult or fast breathing) in the last two weeks for whom care was sought from an appropriate provider, i.e., health posts, health centres, hospitals, or private clinics.

Explanatory variables

We used the Euclidian distance and the difference in altitude from the participants' houses to the nearest health facility and household wealth as explanatory variables. Furthermore, we included other socio-demographic variables such as age, sex, parity, region, caregiver's education level, and number of children in the household to adjust for potential confounding in the analyses.

Data management and analysis

Household data on care utilization were linked with the GPS information of the 142 health centres and 164 health posts that served the households. The linking was done using cluster identification. The Euclidian distance from participants' houses to the nearest health facility was calculated using the geographical location of health facilities and households. Thus, we calculated the distance to the nearest health post for child health services (full child immunization and sick child health care utilization) and distance to the nearest health centre for maternal health care services (antenatal care and facility delivery). These distances were divided into tertiles and labelled as short, medium, and long. Similarly, the difference in altitude was estimated using the altitude information of both the households and the health facility and classified as small, medium, and large. Corrections of the GPS readings were done whenever the reading erroneously was positioned outside the study areas. If that was the case, we adjusted by considering the nearest GPS reading captured. To estimate household wealth, we constructed wealth index using 15 household assets and characteristics. We used principal component analysis (PCA) to construct the wealth index and divided it into tertiles (19).

For peer review only - http://bmjopen.bmj.com/site/about/guidelines.xhtml

We first analysed the prevalence of the outcomes variables and graphically displayed the relation between distance, the difference in altitude, household wealth and facility visits for antennal care, facility delivery, child immunization, and sick child services utilization. After that, we used two-level mixed-effects logistic regression modelling to examine the associations with individual-level variables (wealth, number of children in the household, sex, age, parity, and educational status), group-level variables (region, distance, and the difference in altitude) and the interactions between the two. We analysed the crude and adjusted odds ratios with 95% confidence intervals. As individual observations were grouped within the cluster specifically by geography (region, distance to the health facility and their altitude), the regression models we used accounted for confounding and clustering effects. Considering both the distance as well as the difference in altitude would yield a good estimate of the extent of space between the health facilities and the households. The model quality was assessed using Akaki Information Criteria (AIC) and Intracluster Correlation Coefficient. The significance level was set at a P-value of less than 5%. All analyses were done using STATA 15 (Stata Corporation, College Station, Texas).

Patient and public involvement

Patients or the public were not involved in the design or conduct, or reporting or dissemination plans of this research.

Ethics approval and consent to participate

We obtained ethical approval from the London School of Hygiene & Tropical Medicine, London, UK (Ethics Ref 16117), the Ethiopian Public Health Institute (Ethics Ref 613/52), and the University of Gondar (O/V/P/RCS/05/214/2018). During the data collection, written informed consent was obtained from household heads and women, and assent from minors (age 13-18).

RESULTS

Characteristics of the study participants

Out of 11,877 women aged 13-49, 957 had given birth during 12 months before the survey. Of these, we linked 766 women (80%) with the geographic location data of the 142 nearest health centres, since antenatal and delivery services were provided at the health centre level or above (**Table 1**). We collected

For peer review only - http://bmjopen.bmj.com/site/about/guidelines.xhtml

information on 5,786 children from 2 to 59 months of age. Of these, we linked data on 5,285 (91%) children with the geographic location of 164 nearest health posts. We estimated the Euclidian distance (median: 9.7, IQR:5.2-14.4 kilometre) and the difference in altitude (median: 54, IQR:24-1312 meter from the participants' houses to the nearest health facility.

Table 1: Number of women and children with data linked to the health facility Geographical Positioning System information that was used to calculate distance and difference in altitude between home and health facility

Group	Number of cases	Number of health facilities linked[1]	Cases linked[2]
Children 2-59 months	5787	164 health posts	5285 (91%)
Children 12-23 months	992	164 health posts	912 (92%)
Women 13-49 years	957	142 health centres	766 (80%)

[1]Number of health facilities assessed in the study area that were supposed to provide service to the study population
[2]Number of study participants whose data were linked to the health facility location to calculate the distance and difference in altitude between the household and the health facility

Utilization of maternal health services

Of the 766 mothers who had given birth in the previous 12 months before the survey, 39% (95% CI: 35-42) had attended antenatal care four or more times. The proportion of women who had delivered at a health facility was 55% (95% CI: 51-58). There was an inverse relationship between distance, the difference in attitude, and antenatal care or facility delivery (**Figure 2**). There was an increased proportion of prenatal visits and the percentage of facility deliveries with an increasing wealth index.

Utilization of child health services

Among the 912 children in the age group 12-23 months, 36% (95% CI: 33-39) were fully immunised. The two-week morbidity with common childhood illnesses, i.e., fever, diarrhoea, or suspected pneumonia (cough with fast or difficult breathing), was assessed among 5,285 children from 2 to 59 months of age. Of these, 9.4% (95% CI: 8.6 -10.2) were found to have been sick. The proportion of children who sought care from an appropriate provider, such as health post, health centre, hospital, or private clinics, was 35% (95% CI: 31-39). There was no clear pattern in the associations between distance, the difference in altitude, wealth index, and child immunizations or utilization of services for sick children (**Figure 2**).

Determinants of maternal and child health services utilization

For peer review only - http://bmjopen.bmj.com/site/about/guidelines.xhtml

The multilevel regression analysis, after adjusting for age, parity, education, and region showed that women living with higher difference in altitude were less likely to have attended antenatal care four or more times (AOR=0.46; 95% CI: 0.29-0.74) and to have delivered at a health facility (AOR=0.33; 95% CI: 0.19-0.59) ~~Table 2.~~ Women living at a long distance from health facilities were less likely to have delivered at health facility (AOR=0.57; 95% CI: 0.34-0.96). Women from the wealthier household were more likely to have received antenatal care four or more times (AOR=1.67; 95% CI: 1.02-2.75) and delivered at a health facility (AOR=3.70; 95% CI: 2.11-6.47) than their counterparts in the lower wealth tertiles (Table 2).

Table 2: Determinants of antenatal care four or more times and facility delivery in four regions of Ethiopia, 2019.

Characteristic	Category	Antenatal care four or more visits		Institutional delivery	
		**OR (95% CI)**	**AOR (95% CI)**	**OR (95% CI)**	**AOR (95% CI)**
**Household and individual-level characteristics**					
Wealth tertiles	Low	Ref.	Ref.	Ref.	Ref.
	Middle	1.40(0.90,2.19)	1.15(0.74,1.81)	2.48(1.54,4.01)	1.61(0.99,2.60)
	Upper	2.50(1.55,4.02)	1.67(1.02,2.75)	6.85(3.87,12.15)	3.70(2.11,6.48)
Age	<20	Ref.	Ref.	Ref.	Ref.
	21-25	1.84(1.01,3.37)	1.52(0.80,2.89)	0.62(0.34,1.17)	0.72(0.36,1.43)
	26-30	2.33(1.29,4.22)	1.74(0.85,3.53)	0.71(0.38,1.32)	0.99(0.45,2.15)
	31-35	1.99(1.02,3.91)	1.51(0.68,3.40)	0.85(0.41,1.76)	1.35(0.54,3.34
	36-49	1.97(0.90,4.28)	1.42(0.56,3.61)	0.78(0.33,1.85)	1.34(0.47,3.82)
Educational level	No school	Ref.	Ref.	Ref.	Ref.
	Schooling	1.12(0.79,1.60)	1.18(0.80,1.74)	1.53(1.02-2.29)	1.26(0.81,1.96)
Parity	One	Ref.	Ref.	Ref.	Ref.
	Two	0.92(0.51,1.68)	1.01(0.55,1.87)	0.69(0.34,1.41)	0.94(0.46,1.93)
	Three	0.69(0.37,1.30)	0.77(0.39,1.52)	0.37(0.18,0.74)	0.48(0.22,1.05)
	Four	1.35(0.74,2.47)	1.44(0.73,2.84)	0.41(0.20,0.84)	0.62(0.28,1.40)
	≥Five	0.96(0.56,1.62)	1.01(0.56,2.15)	0.38(0.20,0.71)	0.47(0.21,1.03)
**Group-level characteristics**					
Region	Tigray	Ref.	Ref.	Ref.	Ref.
	Amhara	0.93(0.49,1.70)	0.75(0.41,1.36)	1.99(0.91,4.39)	1.35(0.66,2.77)
	Oromia	0.23(0.13,0.45)	0.22(0.12,0.41)	0.13(0.06,0.28)	0.13(0.06,0.26)
	SNNP	1.48(0.66,3.35)	1.05(0.49,3.34)	2.32(0.80,6.75)	1.26(0.48,3.34)
Distance to health facility tertiles	Short	Ref.	Ref.	Ref.	Ref.
	Medium	1.06(0.69,1.64)	1.01(0.67,1.54)	0.87(0.52,1.44)	0.90(0.56,1.47)
	Long	0.75(0.46,1.22)	0.77(0.49,1.19)	0.51(0.29,0.90)	0.57(0.34,0.96)
Difference in altitude tertiles[1]	Small	Ref.	Ref.	Ref.	Ref.
	Medium	0.78(0.49,1.22)	0.85(0.55,1.29)	0.59(0.34,1.02)	0.60(0.36,1.01)
	Great	0.48(0.28,0.83)	0.46(0.29,0.74)	0.39(0.19,0.79)	0.34(0.19,0.59)
ICC for the null model (%)			27%		49%
ICC for the final model (%)			8%		13%

[1]Difference in altitude between household and nearest health centre

For peer review only

There was no clear pattern in the associations between distance from home to health post, the difference in altitude, or household wealth and child immunization and sick childcare utilization (Table 3). There was no indication of socio-economic inequity in coverage of immunization or utilization of services for the sick child. The medium distance stratum showed a lower coverage.

Table 3: Determinants of child immunization and sick child services utilization in four regions of Ethiopia; 2019.

Variable	Category	Full immunization 12-23 months		Sick child care utilization 2-59 months	
		**OR (95% CI)**	**AOR (95% CI)**	**OR (95% CI)**	**AOR (95% CI)**
**Household and individual-level characteristics**					
Wealth tertiles	Low	Ref.	Ref.	Ref.	Ref.
	Middle	0.91(0.52, 1.62)	0.75(0.42,1.34)	0.73(0.39,1.37)	0.71(0.38,1.33)
	Upper	1.58(0.91,2.77)	1.15(0.61,2.17)	1.02(0.55,1.91)	0.89(0.42,1.88)
Sex	Girl	Ref.	Ref.	Ref.	Ref.
	Boy	1.15(0.84,1.57)	1.18(0.85,1.63)	1.11(0.73,1.66)	1.02(0.67,1.55)
Number of under-five children in the household	One child	Ref.	Ref.	Ref.	Ref.
	≥Two children	1.10(.79, 1.54)	1.21(.86,1.72)	1.15(0.74,1.80)	1.33(0.83,2.14)
**Group-level characteristics**					
Region	Tigray	Ref.	Ref.	Ref.	Ref.
	Amhara	1.32(0.72,2.42)	1.23(0.62,2.45)	2.07(1.03,4.13)	2.26(1.02,5.03)
	Oromia	0.49(0.25,0.94)	0.49(0.25,0.95)	2.87(1.42,5.86)	3.09(1.54,6.19)
	SNNP	0.68(0.30,1.52)	0.55(0.23,1.34)	2.04(0.90,4.62)	2.55(1.07,6.09)
Distance to health facility tertiles	Short	Ref.	Ref.	Ref.	Ref.
	Medium	0.69(0.46,1.04)	0.64(0.41,0.98)	0.49(0.29,0.82)	0.50(0.29,0.85)
	Long	0.84(0.55,1.28)	0.82(0.53,1.27)	0.76(0.44,1.29)	0.76(0.44,1.29)
Difference in altitude tertiles[1]	Small	Ref.	Ref.	Ref.	Ref.
	Medium	1.00(0.66,1.47)	0.99(0.65,1.51)	0.88(0.54,1.44)	1.02(0.61,1.70)
	Great	0.85(0.54,1.35)	0.89(0.55,1.42)	0.48(0.28,0.82)	0.59(0.34,1.01)
ICC for the null model (%)			24%		18%
ICC for the final model (%)			19%		8%

[1]Difference in altitude between household and nearest health facility

DISCUSSION

We have shown that long distance to the health centre, a more significant difference in altitude between home and facility, and a lower socio-economic group were all associated with a smaller proportion of women delivering at a health facility. Similarly, those living with a considerable difference in altitude and belonging to a lower socio-economic group were less likely to have attended antenatal care four times or

For peer review only - http://bmjopen.bmj.com/site/about/guidelines.xhtml

more. However, distance, altitude, or socio-economic status were neither associated with coverage of child immunization nor with care-seeking for sick children.

An estimated 39% of the pregnant women in the current study had attended antenatal care four or more times. This proportion was higher than the corresponding result reported from the same study areas two years earlier, which showed coverage at 30% (10). It was a bit lower than the national average, based on the Demographic and Health Survey 2019, which was 43% (20). This increase could reflect the government's efforts towards universal health coverage (21). In contrast to the current study, in an analysis of the 2016 Ethiopian Demographic and Health Survey, distance to the health centre was associated with four or more antenatal care visits (7). Furthermore, in an analysis of data from Demographic and Health Surveys in five East African counties 2010-2014 showed that geographical accessibility of health facilities had a strong influence on maternal health care utilization (22). The study employed cost-distance analysis based on the health facilities' position and difficulties in traversing the surface of each 300x300 m cell in the study areas. The analysis included the slopes based on elevation measurements as well as the distance from home to the health facility. These findings imply that efforts climbing up and down the hills were barriers to accessing these services. A similar result was shown regarding the influence of household wealth on antenatal care use and facility delivery in the previous study in the same study area (19).

We found that more than half of the study women had delivered at a health facility, which was similar to the coverage (48%) reported by the 2019 Ethiopian Demographic Health Survey (20). In line with the current study, there was also a marked wealth-based inequity in the utilization of antenatal care four or more times and facility delivery in a study performed in the same areas two years earlier (19). Reviews of similar Ethiopian studies (23), research from sub-Saharan Africa countries (24) and East African countries (22), as well as from a range of low-income countries (25) point at considerable regional variation, social inequities in the utilization of facility delivery services, and the importance of topography

and distance from home to health centres and hospitals. A study from Kenya which attempted to disentangle distance to facility and quality of care indicated that pregnant women bypassed possible places to deliver, preferring to give birth at a facility that provided better-quality care (26). Furthermore, an analysis from 81 low and middle-income countries suggested that closing the quality gap would enhance the use of services (27). Such evidence suggest that the problem with distance to the health facility may be less important if the services offered maintain a high quality. In some areas, it may be motivated to consider outreach antenatal clinics organized by the health centres. The problems with distance to facilities for delivery services may be managed by schemes to organize transport or manage associated costs or by improving maternal waiting homes.

In terms of utilization of child health services, thirty-six percent of children aged 12-23 months had received all basic immunizations. This low level was similar to the national estimate (38%) from 2019 Ethiopian mini-Demographic and Health Survey (18). A study two years earlier in the same rural areas as the current study showed an equitable distribution of full child immunization across different socio-economic groups (19). This relative equity in vaccination coverage could be explained by the rural outreach services, organized from the health posts, where eligible children were summoned to vaccination days in their local villages (19). Our analysis showed that the medium tertile of distance to health post was linked to lower immunization coverage and sick child healthcare utilization. This may be a random error, given the lack of consistency across distance strata. If true, it may be a reflection of the geographic distribution of outreach services. A study in Ethiopia's hard-to-reach areas, distance to the health facility influenced full child immunization coverage in addition to the mother's education, utilization antenatal delivery services and child delivered at the health facility (28). However, in an analysis of the 2016 Demographic and Health Survey, full child immunization showed significant variation between groups characterized by geographic, socioeconomic, and maternal factors with pro-advantaged bias (29). The immunizations are given at the health post and partly through outreach services in the villages. This

combination of facility-based and outreach services may theoretically not only increase coverage but also enhance equity (30,31).

One-third of the children 2-59 months, who had been sick in the two weeks before the survey, had sought care from health posts, health centres, hospitals or private clinics which is in line with the national estimates of the utilization of health services for sick children (32). Our results showed no association between care-seeking for sick children and distance to the nearest health centre, difference in altitude and socio-economic status. During the last decade, Ethiopia has scaled up the integrated Community Case Management of common childhood illnesses provided by salaried health extension workers at the health posts (33). The availability of community-level primary care services at health posts close to home, offering child health services, could partly explain the relative equity in utilization. Contrary to this, the 2016 nationally representative Demographic Health Survey showed inequities in care-seeking for common childhood illnesses between groups defined by geographic region, mother's education, or household wealth (32). The Ethiopian government has expanded maternal, neonatal, and child health services in the country (34,35). Further efforts are needed not only to expand these services but also to increase the quality of care (36,37) to reach the goal of Universal Health Coverage (21).

The difference in the influence of distance and difference in altitude between maternal and child health service may be related to the differences in service provision. The child health services, mainly managed by the health posts, include outreach activities with immunization and home-based care. Women had to reach the more distant health centres to receive antenatal and delivery care.

The sample for this study was drawn from intervention and comparison areas for the evaluation of the Optimization of the Health Extension Program intervention, which was implemented in selected districts of the four most populous Ethiopian regions (15). Thus, the results may not be generalizable to the population at country level. We have, however, reasons to believe that the selected districts are typical for agrarian areas of these major Ethiopian regions. Previously published studies have focused on one or a few components of maternal and child care utilization (38,39), but we assessed the major service

coverages, i.e., antenatal care visits, facility delivery, immunization, and utilization of health services for sick children. In cross-sectional studies like this, there is a risk of biased reporting of care utilization. To reduce that risk, we limited the maternal services indicators to the last 12 months. The immunization coverage adhered to the methodology of the Demographic and Health Surveys, including children aged 12-23 months. Similarly, we followed the common practice of limiting common childhood illnesses and care-seeking to a two-weeks recall.

We assessed the straight-line distance between home and the nearest health facility. In reality, it may not be possible to travel in a straight line to a specific location and the route from home to the nearest health facility may be longer than the straight line as one has to avoid obstacles such as a river or a steep slope. In such cases, using cost-distance measurements had given a more realistic measurement. Some home-to-facility distance data were missing, especially regarding the maternal indicators, i.e., the distance from home to the health centre. The reason was missing GPS coordinates of the health centre the family was supposed to use. We have no reason to believe that these omissions introduced any biased estimates. We added the difference in altitude as a potentially important dimension in care utilization. In Ethiopia, with its mountains and valleys, the difference in altitude expresses some of the time and exact distance by road or path from home to the health facility.

We assessed altitude by GPS data that may not always be accurate. However, the error is usually of little importance; in the range from 10 to 20 meters. The socio-economic status was based on assets and characteristics of the household in the way that is practised in the Demographic and Health Surveys. This characterization represents accumulated wealth and resources but does not directly represent the economic strength of the household to cover costs for care-seeking. Given the relative imprecision of the included determinants, we categorized these data into tertiles.

We used multi-level regression modelling that included the determinants, the outcome, and potential confounding factors at the cluster, household, and individual levels. The regression models accounted for confounding and clustering effects.

CONCLUSION

The topography, i.e., the distance and difference in altitude of the areas, and socio-economic status of the household affected the utilization of maternal health services. We did not find such inequities in the utilization of child health services, although coverage was on a low level. The strive for universal health coverage in Ethiopia will require not only increased coverage but also targeted efforts to address the geographic and social differentials in care utilization.

For peer review only

For peer review only - http://bmjopen.bmj.com/site/about/guidelines.xhtml

List of abbreviations

AOR	Adjusted Odds Ratio
GPS	Geographical Positioning System
ICC	Intra-class Correlation Coefficient

Declaration

Funding

The study was funded by a grant to the London School of Hygiene & Tropical Medicine from the Bill and Melinda Gates Foundation (OPP1132551)

Availability of data

Data could be available at a reasonable request to the corresponding author, Atkure Defar, atkuredefar@gmail.com

Authors' contributions

The study was conceived by AD with inputs from LÅP and KA. AD, LÅP, KA, ZT and YO contributed in the acquisition, analysis, or interpretation of data. AD prepared the first draft of the manuscript. LÅP, YO, ZT and KA contributed to the interpretation of results and revision of the manuscript. All authors read and commented on the manuscript and approved the final version of the paper.

Consent for publication

Not applicable

Competing interests

None declared

Acknowledgements

The authors would like to thank the University of Gondar for the support provided to the project. We are also grateful to the regional health bureaus and district health offices in the study areas for their smooth

For peer review only

and kind cooperation. Special thanks to all field staff for their excellent fieldwork and to the study participants for their kind cooperation in interviews and data collection.

REFERENCES

1. World Health Organization, The World Bank. Tracking Universal Health Coverage - First Global Monitoring Report. Geneva, WHO; 2015.
2. Boerma T, Requejo J, Victora CG, Amouzou A, George A, Agyepong I, et al. Countdown to 2030: tracking progress towards universal coverage for reproductive, maternal, newborn, and child health. Lancet [Internet]. 2018 Apr 14;391(10129):1538–48. Available from: https://doi.org/10.1016/S0140-6736(18)30104-1
3. Schaaf H, Hesse G. Schwindelerkrankungen im kindesalter: Erweiterte diagnosemöglichkeiten der vestibulären komponente. Vol. 79, Padiatrische Praxis. 2012. p. 253–65.
4. Memirie ST, Verguet S, Norheim OF, Levin C, Johansson KA. Inequalities in utilization of maternal and child health services in Ethiopia: The role of primary health care Health systems and services in low and middle income settings. BMC Health Serv Res [Internet]. 2016;16(1):1–8. Available from: http://dx.doi.org/10.1186/s12913-016-1296-7
5. Kassile T, Lokina R, Mujinja P, Mmbando BP. Determinants of delay in care seeking among children under five with fever in Dodoma region, central Tanzania: a cross-sectional study. Malar J. 2014 Sep;13:348.
6. Lohela TJ, Campbell OMR, Gabrysch S. Distance to Care, Facility Delivery and Early Neonatal Mortality in Malawi and Zambia. PLoS One. 2012;7(12).
7. Tegegne TK, Chojenta C, Getachew T, Smith R, Loxton D. Antenatal care use in Ethiopia: A spatial and multilevel analysis. BMC Pregnancy Childbirth. 2019;19(1):399.
8. Tadele N, Lamaro T. Utilization of institutional delivery service and associated factors in Bench Maji zone, Southwest Ethiopia: community based, cross sectional study. BMC Health Serv Res. 2017;17(1):1–10.
9. Jimma R, District H, Zegeye K, Gebeyehu A, Melese T. Primary Health Care : Open Access The Role of Geographical Access in the Utilization of Institutional Delivery. Prim Heal care. 2014;4(1):1–6.
10. Defar A, Okwaraji YB, Tigabu Z, Persson LÅ, Alemu K. Geographic differences in maternal and child health care utilization in four Ethiopian regions; a cross-sectional study. Int J Equity Health. 2019;18(1):173.
11. Shiferaw S, Spigt M, Seme A, Amogne A, Skrøvseth S, Desta S, et al. Does proximity of women to facilities with better choice of contraceptives affect their contraceptive utilization in rural Ethiopia? PLoS One. 2017;12(11).
12. Tanou M, Kamiya Y. Assessing the impact of geographical access to health facilities on maternal healthcare utilization: Evidence from the Burkina Faso demographic and health survey 2010. BMC Public Health. 2019;19(1):1–8.
13. Nesbitt RC, Lohela TJ, Soremekun S, Vesel L, Manu A, Okyere E, et al. The influence of distance and quality of care on place of delivery in rural Ghana. Sci Rep [Internet]. 2016;6(August):1–8.

For peer review only - http://bmjopen.bmj.com/site/about/guidelines.xhtml

Available from: http://dx.doi.org/10.1038/srep30291

14. Sarrassat S, Meda N, Badolo H, Ouedraogo M, Some H, Cousens S. Distance to care, care seeking and child mortality in rural Burkina Faso: findings from a population-based cross-sectional survey. Trop Med Int Health. 2019 Jan;24(1):31–42.

15. Berhanu D, Okwaraji YB, Belayneh AB, Lemango ET, Agonafer N, Birhanu BG et al. Protocol for the evaluation of a complex intervention aiming at increased utilization of primary child health services in Ethiopia. A before and after study in intervention and comparison areas. BMC Health Serv Res. 2020;20(1):339–12.

16. CSA. Population Census Commission ii [Internet]. Mortality. 2007. Available from: http://www.csa.gov.et/surveys/Population and Housing census/ETH-pop-2007/survey0/data/Doc/Reports/National_Statistical.pdf

17. Central Statistical Agency and ICF International, Findings K. Ethiopia Demographic and Health Survey, 2011 [Internet]. Addis Ababa, Ethiopia; 2011. Available from: Central Statistical Agency (CSA) [Ethiopia] and ICF. 2017. 2016 Ethiopia Demographic and Health Survey Key Findings. Addis Ababa, Ethiopia, and Rockville, Maryland, USA. CSA and ICF

18. Tamirat KS, Sisay MM. Full immunization coverage and its associated factors among children aged 12-23 months in Ethiopia: Further analysis from the 2016 Ethiopia demographic and health survey. BMC Public Health. 2019;19(1):1–7.

19. Wuneh AD, Medhanyie AA, Bezabih AM, Persson LÅ, Schellenberg J, Okwaraji YB. Wealth-based equity in maternal, neonatal, and child health services utilization: A cross-sectional study from Ethiopia. Int J Equity Health. 2019;18(1):1–9.

20. Ethiopian Public Health Institute (EPHI) and ICF. 2019. Mini Demographic and Health Survey 2019: key Indicators. Rockville, Maryland, USA: EPHI and ICF. 2019.

21. Alebachew A, Hatt L, Kukla M. Monitoring and Evaluating Progress towards Universal Health Coverage in Ethiopia. PLoS Med. 2014;11(9):9–11.

22. Ruktanonchai CW, Ruktanonchai NW, Nove A, Lopes S, Pezzulo C, Bosco C, et al. Equality in maternal and newborn health: Modelling geographic disparities in utilisation of care in five East African countries. PLoS One. 2016;11(8):1–17.

23. Kebede A, Hassen K, Teklehaymanot AN. Factors associated with institutional delivery service utilization in Ethiopia. Int J Womens Health. 2016;8:463–75.

24. Moyer CA, Mustafa A. Drivers and deterrents of facility delivery in sub-Saharan Africa: A systematic review. Reprod Health. 2013;10(1):40.

25. Karra M, Fink G, Canning D. Facility distance and child mortality: a multi-country study of health facility access, service utilization, and child health outcomes. Int J Epidemiol. 2017 Jun;46(3):817–26.

26. Escamilla V, Calhoun L, Winston J, Speizer IS. The Role of Distance and Quality on Facility Selection for Maternal and Child Health Services in Urban Kenya. J Urban Health. 2018 Feb;95(1):1–12.

27. Chou VB, Walker N, Kanyangarara M. Estimating the global impact of poor quality of care on

For peer review only

maternal and neonatal outcomes in 81 low- And middle-income countries: A modeling study. PLoS Med. 2019;16(12):1–16.

28. Girmay A, Dadi AF. Full Immunization Coverage and Associated Factors among Children Aged 12-23 Months in a Hard-to-Reach Areas of Ethiopia. Int J Pediatr. 2019;2019:1–8.

29. Geweniger A, Abbas KM. Childhood vaccination coverage and equity impact in Ethiopia by socioeconomic, geographic, maternal, and child characteristics. Vaccine [Internet]. 2020;38(20):3627–38. Available from: https://doi.org/10.1016/j.vaccine.2020.03.040

30. Oyo-Ita A, Cs W, Oringanje C, Ce N, Oduwole O, Mm M. Interventions for improving coverage of childhood immunisation in low- andmiddle-income countries. Cochrane Database Syst Rev [Internet]. 2016;(7). Available from: https://www.ncbi.nlm.nih.gov/pubmed/27394698

31. Utazi CE, Thorley J, Alegana VA, Ferrari MJ, Takahashi S, Metcalf CJE, et al. Mapping vaccination coverage to explore the effects of delivery mechanisms and inform vaccination strategies. Nat Commun [Internet]. 2019;10(1):1–10. Available from: http://dx.doi.org/10.1038/s41467-019-09611-1

32. Central Statistical Agency (CSA) [Ethiopis], ICF. Ethiopia Demographic and Health Survey 2016. Addis Ababa, Ethiopia, and Rockville, Maryland, USA; 2016.

33. Miller NP, Amouzou A, Tafesse M, Hazel E, Legesse H, Degefie T, et al. Integrated Community Case Management of Childhood Illness in Ethiopia : Implementation Strength and Quality of Care. Am J Trop Med Hyg. 2014;91(2):424–34.

34. Byrne A, Hodge A, Jimenez-Soto E, Morgan A. What works? Strategies to increase reproductive, maternal and child health in difficult to access mountainous locations: A systematic literature review. PLoS One. 2014;9(2).

35. Karim AM, Tamire A, Medhanyie AA, Betemariam W. Changes in equity of maternal, newborn, and child health care practices in 115 districts of rural Ethiopia: Implications for the health extension program. BMC Pregnancy Childbirth [Internet]. 2015;15(1):1–11. Available from: http://dx.doi.org/10.1186/s12884-015-0668-z

36. Allen E, Schellenberg J, Berhanu D, Cousens S, Marchant T. Associations between increased intervention coverage for mothers and newborns and the number and quality of contacts between families and health workers: An analysis of cluster level repeat cross sectional survey data in Ethiopia. PLoS One. 2018;13(8):1–13.

37. Defar A, Getachew T, Taye G, Tadele T, Getnet M, Shumet T, et al. Quality antenatal care services delivery at health facilities of Ethiopia, assessment of the structure/input of care setting. BMC Health Serv Res. 2020;20(1):485.

38. Ayalneh AA, Fetene DM, Lee TJ. Inequalities in health care utilization for common childhood illnesses in Ethiopia: evidence from the 2011 Ethiopian Demographic and Health Survey. Int J Equity Health [Internet]. 2017;16(1):67. Available from: http://equityhealthj.biomedcentral.com/articles/10.1186/s12939-017-0561-7

39. Alene M, Yismaw L, Berelie Y, Kassie B. Health care utilization for common childhood illnesses in rural parts of Ethiopia: Evidence from the 2016 Ethiopian demographic and health survey. BMC Public Health. 2019;19(1):1–12.

For peer review only - http://bmjopen.bmj.com/site/about/guidelines.xhtml

Figure legend

Figure 1 Maps showing A) Ethiopia, B) the four study regions, and C) the 46 study districts in the four regions (Graph constructed in ArcGIS 10.4 software using freely available data from the Ethiopian Central Statistics Agency)

Figure 2: The distribution of antenatal care, facility delivery, child immunization and sick childcare utilization by tertiles of the distance and difference in altitude between the household and the nearest health facility, and the wealth index.

For peer review only

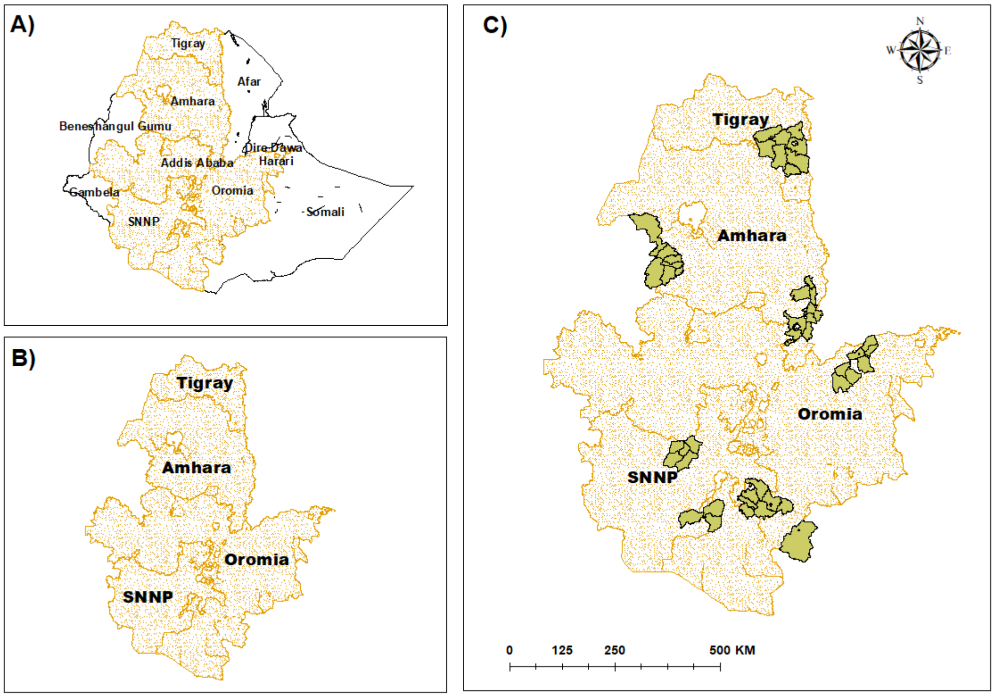

296x210mm (96 x 96 DPI)

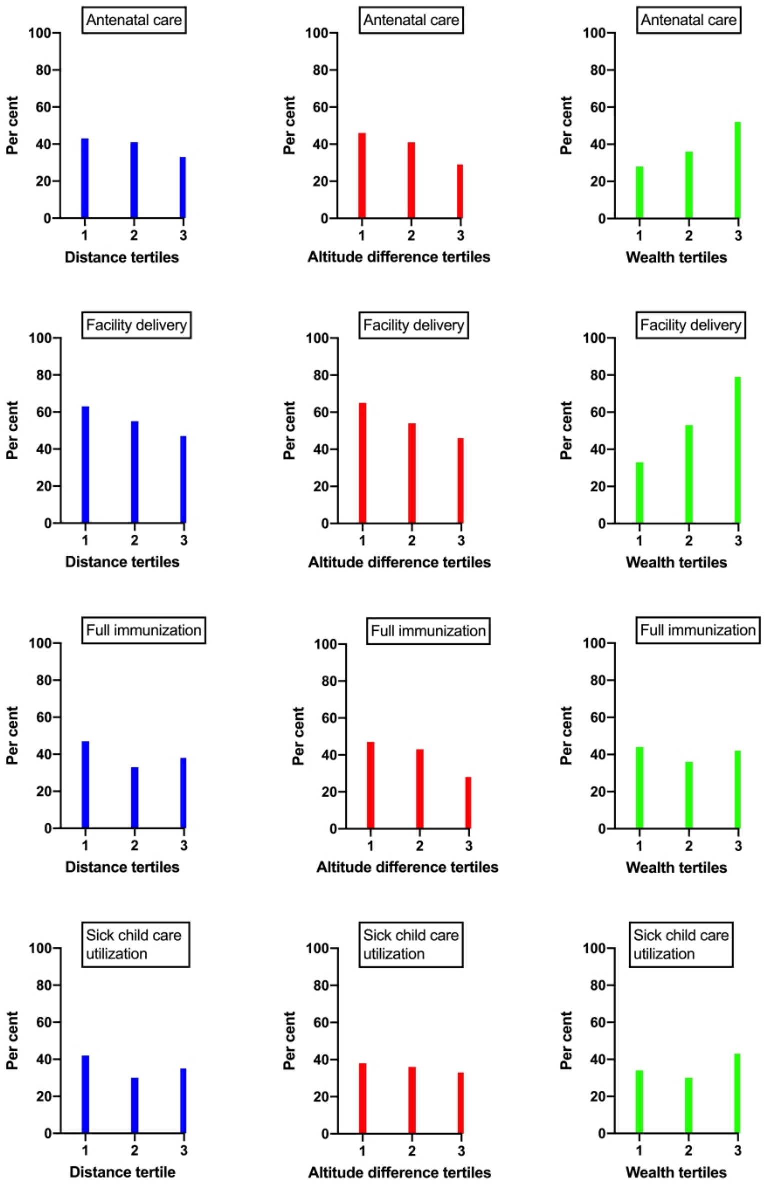

178x269mm (600 x 600 DPI)

STROBE—Checklist of items for a cross-sectional studies

Manuscript section	Item No	Description	Reported ✓	Page #
**Title and abstract**	1	(*a*) Indicate the study's design with a commonly used term in the title or the abstract		
		(*b*) Provide in the abstract an informative and balanced summary of what was done and what was found	✓	2
**Introduction**				
Background/rationale	2	Explain the scientific background and rationale for the investigation being reported	✓	4-5
Objectives	3	State specific objectives, including any prespecified hypotheses	✓	5
**Methods**				
Study design	4	Present key elements of study design early in the paper	✓	6
Setting	5	Describe the setting, locations, and relevant dates, including periods of recruitment, exposure, follow-up, and data collection	✓	6
Participants	6	(*a*) Give the eligibility criteria, and the sources and methods of selection of participants	✓	6-7
Variables	7	Clearly define all outcomes, exposures, predictors, potential confounders, and effect modifiers. Give diagnostic criteria, if applicable	✓	7
Data sources/ measurement	8*	For each variable of interest, give sources of data and details of methods of assessment (measurement). Describe comparability of assessment methods if there is more than one group	✓	*2 & 7*
Bias	9	Describe any efforts to address potential sources of bias	✓	4
Study size	10	Explain how the study size was arrived at		
Quantitative variables	11	Explain how quantitative variables were handled in the analyses. If applicable, describe which groupings were chosen and why	✓	6-7
Statistical methods	12	(*a*) Describe all statistical methods, including those used to control for confounding	✓	8
		(*b*) Describe any methods used to examine subgroups and interactions		
		(*c*) Explain how missing data were addressed	✓	8
		(*d*) If applicable, describe analytical methods taking account of sampling strategy	✓	8
		(*e*) Describe any sensitivity analyses		
**Results**				
Participants	13*	(a) Report numbers of individuals at each stage of study—eg numbers potentially eligible, examined for eligibility, confirmed eligible, included in the study, completing follow-up, and analysed	✓	9-10
		(b) Give reasons for non-participation at each stage		

For peer review only

		(c) Consider use of a flow diagram		
Descriptive data	14*	(a) Give characteristics of study participants (eg demographic, clinical, social) and information on exposures and potential confounders	✓	10
		(b) Indicate number of participants with missing data for each variable of interest	✓	10
Outcome data	15*	Report numbers of outcome events or summary measures	✓	10
Main results	16	(*a*) Give unadjusted estimates and, if applicable, confounder-adjusted estimates and their precision (eg, 95% confidence interval). Make clear which confounders were adjusted for and why they were included	✓	11
		(*b*) Report category boundaries when continuous variables were categorized	✓	10
		(*c*) If relevant, consider translating estimates of relative risk into absolute risk for a meaningful time period		
Other analyses	17	Report other analyses done—eg analyses of subgroups and interactions, and sensitivity analyses		
**Discussion**				
Key results	18	Summarise key results with reference to study objectives	✓	12-13
Limitations	19	Discuss limitations of the study, taking into account sources of potential bias or imprecision. Discuss both direction and magnitude of any potential bias	✓	4
Interpretation	20	Give a cautious overall interpretation of results considering objectives, limitations, multiplicity of analyses, results from similar studies, and other relevant evidence	✓	12-14
Generalisability	21	Discuss the generalisability (external validity) of the study results	✓	14
**Other information**				
Funding	22	Give the source of funding and the role of the funders for the present study and, if applicable, for the original study on which the present article is based	✓	16

*Give information separately for exposed and unexposed groups.

Note: An Explanation and Elaboration article discusses each checklist item and gives methodological background and published examples of transparent reporting. The STROBE checklist is best used in conjunction with this article (freely available on the Web sites of PLoS Medicine at http://www.plosmedicine.org/, Annals of Internal Medicine at http://www.annals.org/, and Epidemiology at http://www.epidem.com/). Information on the STROBE Initiative is available at www.strobe-statement.org.

For peer review only

Distance, difference in altitude, and socio-economic determinants of utilization of maternal and child health services in Ethiopia: a geographic and multilevel modelling analysis

Journal:	*BMJ Open*
Manuscript ID	bmjopen-2020-042095.R2
Article Type:	Original research
Date Submitted by the Author:	23-Dec-2020
Complete List of Authors:	Defar, Atkure ; Ethiopian Public Health Institute; University of Gondar College of Medicine and Health Sciences, Institute of Public Health, Department of Epidemiology and Biostatistics<br>Okwaraji, Yemisrach; London School of Hygiene and Tropical Medicine Faculty of Infectious and Tropical Diseases, Department of Disease Control; Ethiopian Public Health Institute<br>Tigabu, Zemene; University of Gondar College of Medicine and Health Sciences, Department of Paediatrics and Child Health<br>Persson, Lars; London School of Hygiene and Tropical Medicine Faculty of Infectious and Tropical Diseases, Department of Disease Control; Ethiopian Public Health Institute<br>Alemu, Kassahun ; University of Gondar College of Medicine and Health Sciences, Institute of Public Health, Department of Epidemiology and Biostatistics
**Primary Subject Heading**:	Public health
Secondary Subject Heading:	Health services research, Public health, Epidemiology
Keywords:	HEALTH SERVICES ADMINISTRATION & MANAGEMENT, Community child health < PAEDIATRICS, PUBLIC HEALTH

I, the Submitting Author has the right to grant and does grant on behalf of all authors of the Work (as defined in the below author licence), an exclusive licence and/or a non-exclusive licence for contributions from authors who are: i) UK Crown employees; ii) where BMJ has agreed a CC-BY licence shall apply, and/or iii) in accordance with the terms applicable for US Federal Government officers or employees acting as part of their official duties; on a worldwide, perpetual, irrevocable, royalty-free basis to BMJ Publishing Group Ltd ("BMJ") its licensees and where the relevant Journal is co-owned by BMJ to the co-owners of the Journal, to publish the Work in this journal and any other BMJ products and to exploit all rights, as set out in our licence.

The Submitting Author accepts and understands that any supply made under these terms is made by BMJ to the Submitting Author unless you are acting as an employee on behalf of your employer or a postgraduate student of an affiliated institution which is paying any applicable article publishing charge ("APC") for Open Access articles. Where the Submitting Author wishes to make the Work available on an Open Access basis (and intends to pay the relevant APC), the terms of reuse of such Open Access shall be governed by a Creative Commons licence – details of these licences and which Creative Commons licence will apply to this Work are set out in our licence referred to above.

Other than as permitted in any relevant BMJ Author's Self Archiving Policies, I confirm this Work has not been accepted for publication elsewhere, is not being considered for publication elsewhere and does not duplicate material already published. I confirm all authors consent to publication of this Work and authorise the granting of this licence.

For peer review only

Distance, difference in altitude, and socio-economic determinants of utilization of maternal and child health services in Ethiopia: a geographic and multilevel modelling analysis

Atkure Defar[1, 4*], Yemisrach B. Okwaraji[1, 3], Zemene Tigabu[2], Lars Åke Persson[1, 3], Kassahun Alemu[4]

*Corresponding author:

Atkure Defar, MPH, MBA, Email: atkuredefar@gmail.com

ORCID, https://orcid.org/0000-0001-9435-2135

Co-authors:

Yemisrach Okwaraji, PhD, Email, yemisrach.okwaraji@lshtm.ac.uk

ORCID, https://orcid.org/0000-0002-4541-2856

Zemene Tigabu MD/Paediatrician, Email: zemene.tigabu@gmail.com

ORCID, https://orcid.org/0000-0001-6040-032X

Prof. Lars Åke Persson; MD, PhD, Email: Lars.Persson@lshtm.ac.uk

ORCID, https://orcid.org/0000-0003-0710-7954

Kassahun Alemu Gelaye, PhD, Email: kassalemu@gmail.com

ORCID, https://orcid.org/0000-0002-8360-0292

Affiliation

[1]Ethiopian Public Health Institute, Addis Ababa, Ethiopia

[2]Department of Paediatrics and Child Health, School of Medicine, College of Medicine and Health Sciences, University of Gondar, Gondar, Ethiopia

[3]London School of Hygiene and Tropical Medicine, London, UK

[4]Department of Epidemiology and Biostatistics, Institute of Public Health, College of Medicine and Health Sciences, University of Gondar, Gondar, Ethiopia

Word count 3372

For peer review only

ABSTRACT

Objective We assessed whether geographic distance and difference in altitude between home to health facility and household socio-economic status were associated with utilization of maternal and child health services in rural Ethiopia.

Design Household and health facility surveys were conducted from December 2018 to February 2019.

Setting Fortysix districts in the Ethiopian regions Amhara, Oromia, Tigray, and Southern Nations, Nationalities, and Peoples.

Participants A total of 11,877 women aged 13-49 years and 5,786 children aged 2-59 months were included.

Outcome measures The outcomes were four or more antenatal care visits, facility delivery, full child immunization, and utilization of health services for sick children. A multilevel analysis was carried out with adjustments for potential confounding factors.

Results Overall, 39% (95% CI: 35-42) women had attended four or more antenatal care visits, and 55% (95%CI: 51-58) delivered at health facilities. One in three (36%, 95%CI: 33-39) of children had received full-immunizations and 35% (95% CI: 31-39) of sick children used health services. A long distance (AOR=0.57; 95% CI 0.34-0.96), and larger difference in altitude (AOR=0.34; 95%CI 0.19-0.59) were associated with fewer facility deliveries. Larger difference in altitude was associated with a lower proportion of antenatal care visits (AOR=0.46; 95%CI: 0.29-0.74). A higher wealth index was associated with a higher proportion of antenatal care visits (AOR=1.67; 95%CI: 1.02-2.75) and health facility deliveries (AOR=2.11; 95%CI: 2.11-6.48). There was no association between distance, difference in altitude, or wealth index and children being fully immunized or seeking care when they were sick.

Conclusion Achieving universal access to maternal and child health services will require not only strategies to increase coverage but also targeted efforts to address the geographic and socio-economic differentials in care utilization, especially for maternal health.

For peer review only

Keywords: Distance, Altitude, Antenatal care, Facility delivery, Immunization coverage, Sick child services utilization, Ethiopia

Strengths and limitations of this study

- We assessed geographic and social equity across the continuum of maternal and child healthcare, i.e., antenatal care visits, facility deliveries, immunization, and utilization of health services for sick children.
- We used a multi-level regression modelling that included the determinants, the outcome, and potential confounding factors at the cluster, household, and individual levels accounting for confounding and clustering effects.
- We limited reporting of maternal services utilisation to the last 12 months and care-seeking for common childhood illnesses to a two-week recall period to reduce recall bias.
- In addition to the straight-line distance between home and the nearest health facility, we assessed the difference in altitude between home and the nearest health facility as a potentially important dimension of distance that could affect care utilization.
- The distance measured between household and health facility does not entirely reflect the actual distance on the ground.

INTRODUCTION

The global goal of Universal Health Coverage is a significant challenge since half of the world's population does not have access to essential health services (1). The Sustainable Development Goals stresses the need for accelerating the ambitions to reach the targets for improving maternal, newborn, and child health (2). The World Health Organization, with its partners, has developed policies and programs aiming to promote health equity across and within nations (3). Utilization of maternal, newborn, and child

health services has increased. However, inequities by geographic location and socioeconomic status are challenges in the efforts towards universal health coverage (4,5).

Studies from low-income African countries have indicated a strong association between geographic accessibility to health facilities and the use of maternal and child health services (6). In Ethiopia, reports have described geographical inequality. Distance measured as a straight line and walking time was associated with attendance to antenatal care and facility delivery (7–9). The topography of Ethiopia, characterized by difficult steep terrain with high hills, valleys and long distances from home to health facilities, has contributed to inequalities in the use of maternal and child health services (10). In addition, studies in Ethiopia and other African countries have shown that distance to health facilities was negatively associated with the use of family planning (11), antenatal care (12), facility deliveries (13), and care-seeking for sick children (14)

To the best of our knowledge, no previous studies have simultaneously analysed the difference in altitude, the distance between household and health facility, as well as socio-economic characteristics and their possible association with utilization of a range of maternal and child health services in Ethiopia. Therefore, this study aimed to analyse if the geographic distance from home to the nearest health facility, the difference in altitude between home and health facility, and socio-economic status were associated with the use of antenatal care, facility delivery, full child immunization, and utilization of primary care services for sick children in rural Ethiopia.

METHODS

Study design and setting

We conducted a cross-sectional study in 46 districts of the four most populous Ethiopian regions, namely, Amhara, Oromia, Tigray, and Southern Nations, Nationalities, and Peoples (**Figure 1**). The regions have a population scattered in dispersed settlements, and the primary source of income is farming.

For peer review only

In the Ethiopian health delivery system, the primary health care unit is comprised of five satellite health posts and one health centre. Each health post serves approximately 5,000 people and is staffed by two female Health Extension Workers.

The study was conducted from December 2018 to February 2019. The Ethiopian Public Health Institute, in collaboration with the London School of Hygiene and Tropical Medicine, University of Gondar, Mekelle University, Jimma University, and Hawassa University, conducted the survey.

Sampling

This study used data from an evaluation of the Optimization of Health Extension Program intervention that was implemented in four regions of Ethiopia. This intervention aimed to improve care utilization for sick under-five children (15). The evaluation included baseline and endline surveys, and this study was a secondary analysis of end-line data.

A two-stage stratified cluster sampling was applied in the selected study districts. The first stage used lists of enumeration areas from the 2007 Ethiopian Housing and Population Census as the sampling frame (16). Thereafter, 194 enumeration areas were selected with probability proportional to size (sampling from a finite population in which a size measure is available for each population unit before sampling and where the probability of selecting a unit is proportional to its size.

In the second stage, all households within each enumeration area were listed, and a sampling interval was calculated. A random start number between one and the sampling interval was selected. The households that matched the random start number on the list were chosen as the first household to be included. This process was repeated until the targeted number of 60 households in each cluster was reached. All women aged 13 to 49 years and children under the age of five years, who lived in the selected households, were included in the study. The health posts and the health centres serving the enumeration area were included in the study.

For peer review only

We used a standard sample size formula to calculate the sample size. We estimated a design effect of 1.3, 80% power, and the assumption of a ratio of children less than five years of age per household was considered, based on sampling reported in the Ethiopia DHS 2011 (17). We used the assumption to detect 15% improved childcare utilization in two groups (intervention and comparison) between baseline and end-line surveys. The sample size was estimated to be 6,000 households per group (12,000 in total) to have 80% power to detect differences of 15 percentage points for care-seeking for 2-59 months children. The protocol for the evaluation study was registered with a trial number ISRCTN12040912 and published (15).

Data collection tools

We used a modular questionnaire that was based on the Demographic and Health Survey and similar assessment tools. Information on the antenatal care attendance and place of delivery was collected from all women aged 13-49 who had a live birth in the last 12 months before the survey. Caregivers of children aged 2-59 months were asked whether the child had any illness in the previous two weeks and if they had sought care from an appropriate provider. Caregivers were invited to show the immunization cards and asked additional questions on the different vaccinations if the card was not available. Besides, the questionnaire included information on socio-demographic data, assets, and geographic coordinates of households and health facilities. The coordinates were measured by GPS dongles (ND-100S).

For peer review only

Definition of variables

Outcome variables

We estimated the overall coverage of four selected maternal and child health services. For women, this included 1) the proportion of women aged 13 to 49 with a live birth within the last 12 months before the survey who had attended antenatal care four or more times, and 2) the proportion of women with a live birth within 12 months before the survey who gave birth in a health facility. For the child health services utilization we included: 1) the proportion of children aged 12–23 months who had received full

immunization, which was defined as Bacillus Calmette-Guerin (BCG), three pentavalent vaccinations (diphtheria, tetanus, pertussis, hepatitis B, and Haemophilus influenzae), oral polio vaccine, and one dose of measles vaccine (18), and 2) the proportion of children aged 2–59 months with fever, diarrhoea, or suspected pneumonia (cough and difficult or fast breathing) in the last two weeks for whom care was sought from an appropriate provider, i.e., health posts, health centres, hospitals, or private clinics.

Explanatory variables

We used the Euclidian distance and the difference in altitude from the participants' houses to the nearest health facility and household wealth as explanatory variables. Furthermore, we included other socio-demographic variables such as age, sex, parity, region, caregiver's education level, and number of children in the household to adjust for potential confounding in the analyses.

Data management and analysis

Household data on care utilization were linked with the GPS information of the 142 health centres and 164 health posts that served the households. The linking was done using cluster identification. The Euclidian distance from participants' houses to the nearest health facility was calculated using the geographical location of health facilities and households. Thus, we calculated the distance to the nearest health post for child health services (full child immunization and sick child health care utilization) and distance to the nearest health centre for maternal health care services (antenatal care and facility delivery). These distances were divided into tertiles and labelled as short, medium, and long. Similarly, the difference in altitude was estimated using the altitude information of both the households and the health facility and classified as small, medium, and large. Corrections of the GPS readings were done whenever the reading erroneously was positioned outside the study areas. If that was the case, we adjusted by considering the nearest GPS reading captured. To estimate household wealth, we constructed wealth index using 15 household assets and characteristics. We used principal component analysis (PCA) to construct the wealth index and divided it into tertiles (19).

For peer review only - http://bmjopen.bmj.com/site/about/guidelines.xhtml

We first analysed the prevalence of the outcomes variables and graphically displayed the relation between distance, the difference in altitude, household wealth and facility visits for antennal care, facility delivery, child immunization, and sick child services utilization. After that, we used two-level mixed-effects logistic regression modelling to examine the associations with individual-level variables (wealth, number of children in the household, sex, age, parity, and educational status), group-level variables (region, distance, and the difference in altitude) and the interactions between the two. We analysed the crude and adjusted odds ratios with 95% confidence intervals. As individual observations were grouped within the cluster specifically by geography (region, distance to the health facility and their altitude), the regression models we used accounted for confounding and clustering effects. Considering both the distance as well as the difference in altitude would yield a good estimate of the extent of space between the health facilities and the households. The model quality was assessed using Akaki Information Criteria (AIC) and Intracluster Correlation Coefficient. The significance level was set at a P-value of less than 5%. All analyses were done using STATA 15 (Stata Corporation, College Station, Texas).

Patient and public involvement

Patients or the public were not involved in the design or conduct, or reporting or dissemination plans of this research.

Ethics approval and consent to participate

We obtained ethical approval from the London School of Hygiene & Tropical Medicine, London, UK (Ethics Ref 16117), the Ethiopian Public Health Institute (Ethics Ref 613/52), and the University of Gondar (O/V/P/RCS/05/214/2018). During the data collection, written informed consent was obtained from household heads and women, and assent from minors (age 13-18).

RESULTS

Characteristics of the study participants

Out of 11,877 women aged 13-49, 957 had given birth during 12 months before the survey. Of these, we linked 766 women (80%) with the geographic location data of the 142 nearest health centres, since antenatal and delivery services were provided at the health centre level or above (**Table 1**). We collected

For peer review only - http://bmjopen.bmj.com/site/about/guidelines.xhtml

information on 5,786 children from 2 to 59 months of age. Of these, we linked data on 5,285 (91%) children with the geographic location of 164 nearest health posts. We estimated the Euclidian distance (median: 9.7, IQR:5.2-14.4 kilometre) and the difference in altitude (median: 54, IQR:24-1312 meter from the participants' houses to the nearest health facility.

Table 1: Number of women and children with data linked to the health facility Geographical Positioning System information that was used to calculate distance and difference in altitude between home and health facility

Group	Number of cases	Number of health facilities linked[1]	Cases linked[2]
Children 2-59 months	5787	164 health posts	5285 (91%)
Children 12-23 months	992	164 health posts	912 (92%)
Women 13-49 years	957	142 health centres	766 (80%)

[1]Number of health facilities assessed in the study area that were supposed to provide service to the study population
[2]Number of study participants whose data were linked to the health facility location to calculate the distance and difference in altitude between the household and the health facility

Utilization of maternal health services

Of the 766 mothers who had given birth in the previous 12 months before the survey, 39% (95% CI: 35-42) had attended antenatal care four or more times. The proportion of women who had delivered at a health facility was 55% (95% CI: 51-58). There was an inverse relationship between distance, the difference in attitude, and antenatal care or facility delivery (**Figure 2**). There was an increased proportion of prenatal visits and the percentage of facility deliveries with an increasing wealth index.

Utilization of child health services

Among the 912 children in the age group 12-23 months, 36% (95% CI: 33-39) were fully immunised. The two-week morbidity with common childhood illnesses, i.e., fever, diarrhoea, or suspected pneumonia (cough with fast or difficult breathing), was assessed among 5,285 children from 2 to 59 months of age. Of these, 9.4% (95% CI: 8.6 -10.2) were found to have been sick. The proportion of children who sought care from an appropriate provider, such as health post, health centre, hospital, or private clinics, was 35% (95% CI: 31-39). There was no clear pattern in the associations between distance, the difference in altitude, wealth index, and child immunizations or utilization of services for sick children (**Figure 2**).

Determinants of maternal and child health services utilization

For peer review only - http://bmjopen.bmj.com/site/about/guidelines.xhtml

The multilevel regression analysis, after adjusting for age, parity, education, and region showed that women living with higher difference in altitude were less likely to have attended antenatal care four or more times (AOR=0.46; 95% CI: 0.29-0.74) and to have delivered at a health facility (AOR=0.33; 95% CI: 0.19-0.59) ~~Table 2.~~ Women living at a long distance from health facilities were less likely to have delivered at health facility (AOR=0.57; 95% CI: 0.34-0.96). Women from the wealthier household were more likely to have received antenatal care four or more times (AOR=1.67; 95% CI: 1.02-2.75) and delivered at a health facility (AOR=3.70; 95% CI: 2.11-6.47) than their counterparts in the lower wealth tertiles (Table 2).

For peer review only

Table 2: Determinants of antenatal care four or more times and facility delivery in four regions of Ethiopia, 2019.

Characteristic	Category	Antenatal care four or more visits		Institutional delivery	
		OR (95% CI)	AOR (95% CI)	OR (95% CI)	AOR (95% CI)
**Household and individual-level characteristics**					
Wealth tertiles	Low	Ref.	Ref.	Ref.	Ref.
	Middle	1.40(0.90,2.19)	1.15(0.74,1.81)	2.48(1.54,4.01)	1.61(0.99,2.60)
	Upper	2.50(1.55,4.02)	1.67(1.02,2.75)	6.85(3.87,12.15)	3.70(2.11,6.48)
Age	<20	Ref.	Ref.	Ref.	Ref.
	21-25	1.84(1.01,3.37)	1.52(0.80,2.89)	0.62(0.34,1.17)	0.72(0.36,1.43)
	26-30	2.33(1.29,4.22)	1.74(0.85,3.53)	0.71(0.38,1.32)	0.99(0.45,2.15)
	31-35	1.99(1.02,3.91)	1.51(0.68,3.40)	0.85(0.41,1.76)	1.35(0.54,3.34
	36-49	1.97(0.90,4.28)	1.42(0.56,3.61)	0.78(0.33,1.85)	1.34(0.47,3.82)
Educational level	No school	Ref.	Ref.	Ref.	Ref.
	Schooling	1.12(0.79,1.60)	1.18(0.80,1.74)	1.53(1.02-2.29)	1.26(0.81,1.96)
Parity	One	Ref.	Ref.	Ref.	Ref.
	Two	0.92(0.51,1.68)	1.01(0.55,1.87)	0.69(0.34,1.41)	0.94(0.46,1.93)
	Three	0.69(0.37,1.30)	0.77(0.39,1.52)	0.37(0.18,0.74)	0.48(0.22,1.05)
	Four	1.35(0.74,2.47)	1.44(0.73,2.84)	0.41(0.20,0.84)	0.62(0.28,1.40)
	≥Five	0.96(0.56,1.62)	1.01(0.56,2.15)	0.38(0.20,0.71)	0.47(0.21,1.03)
**Group-level characteristics**					
Region	Tigray	Ref.	Ref.	Ref.	Ref.
	Amhara	0.93(0.49,1.70)	0.75(0.41,1.36)	1.99(0.91,4.39)	1.35(0.66,2.77)
	Oromia	0.23(0.13,0.45)	0.22(0.12,0.41)	0.13(0.06,0.28)	0.13(0.06,0.26)
	SNNP	1.48(0.66,3.35)	1.05(0.49,3.34)	2.32(0.80,6.75)	1.26(0.48,3.34)
Distance to health facility tertiles	Short	Ref.	Ref.	Ref.	Ref.
	Medium	1.06(0.69,1.64)	1.01(0.67,1.54)	0.87(0.52,1.44)	0.90(0.56,1.47)
	Long	0.75(0.46,1.22)	0.77(0.49,1.19)	0.51(0.29,0.90)	0.57(0.34,0.96)
Difference in altitude tertiles[1]	Small	Ref.	Ref.	Ref.	Ref.
	Medium	0.78(0.49,1.22)	0.85(0.55,1.29)	0.59(0.34,1.02)	0.60(0.36,1.01)
	Great	0.48(0.28,0.83)	0.46(0.29,0.74)	0.39(0.19,0.79)	0.34(0.19,0.59)
ICC for the null model (%)			27%		49%
ICC for the final model (%)			8%		13%

[1]Difference in altitude between household and nearest health centre

For peer review only - http://bmjopen.bmj.com/site/about/guidelines.xhtml

There was no clear pattern in the associations between distance from home to health post, the difference in altitude, or household wealth and child immunization and sick childcare utilization (Table 3). There was no indication of socio-economic inequity in coverage of immunization or utilization of services for the sick child. The medium distance stratum showed a lower coverage.

Table 3: Determinants of child immunization and sick child services utilization in four regions of Ethiopia; 2019.

Variable	Category	Full immunization 12-23 months		Sick child care utilization 2-59 months	
		OR (95% CI)	AOR (95% CI)	OR (95% CI)	AOR (95% CI)
**Household and individual-level characteristics**					
Wealth tertiles	Low	Ref.	Ref.	Ref.	Ref.
	Middle	0.91(0.52, 1.62)	0.75(0.42,1.34)	0.73(0.39,1.37)	0.71(0.38,1.33)
	Upper	1.58(0.91,2.77)	1.15(0.61,2.17)	1.02(0.55,1.91)	0.89(0.42,1.88)
Sex	Girl	Ref.	Ref.	Ref.	Ref.
	Boy	1.15(0.84,1.57)	1.18(0.85,1.63)	1.11(0.73,1.66)	1.02(0.67,1.55)
Number of under-five children in the household	One child	Ref.	Ref.	Ref.	Ref.
	≥Two children	1.10(.79, 1.54)	1.21(.86,1.72)	1.15(0.74,1.80)	1.33(0.83,2.14)
**Group-level characteristics**					
Region	Tigray	Ref.	Ref.	Ref.	Ref.
	Amhara	1.32(0.72,2.42)	1.23(0.62,2.45)	2.07(1.03,4.13)	2.26(1.02,5.03)
	Oromia	0.49(0.25,0.94)	0.49(0.25,0.95)	2.87(1.42,5.86)	3.09(1.54,6.19)
	SNNP	0.68(0.30,1.52)	0.55(0.23,1.34)	2.04(0.90,4.62)	2.55(1.07,6.09)
Distance to health facility tertiles	Short	Ref.	Ref.	Ref.	Ref.
	Medium	0.69(0.46,1.04)	0.64(0.41,0.98)	0.49(0.29,0.82)	0.50(0.29,0.85)
	Long	0.84(0.55,1.28)	0.82(0.53,1.27)	0.76(0.44,1.29)	0.76(0.44,1.29)
Difference in altitude tertiles[1]	Small	Ref.	Ref.	Ref.	Ref.
	Medium	1.00(0.66,1.47)	0.99(0.65,1.51)	0.88(0.54,1.44)	1.02(0.61,1.70)
	Great	0.85(0.54,1.35)	0.89(0.55,1.42)	0.48(0.28,0.82)	0.59(0.34,1.01)
ICC for the null model (%)			24%		18%
ICC for the final model (%)			19%		8%

[1]Difference in altitude between household and nearest health facility

DISCUSSION

We have shown that long distance to the health centre, a more significant difference in altitude between home and facility, and a lower socio-economic group were all associated with a smaller proportion of women delivering at a health facility. Similarly, those living with a considerable difference in altitude and belonging to a lower socio-economic group were less likely to have attended antenatal care four times or

For peer review only - http://bmjopen.bmj.com/site/about/guidelines.xhtml

more. However, distance, altitude, or socio-economic status were neither associated with coverage of child immunization nor with care-seeking for sick children.

An estimated 39% of the pregnant women in the current study had attended antenatal care four or more times. This proportion was higher than the corresponding result reported from the same study areas two years earlier, which showed coverage at 30% (10). It was a bit lower than the national average, based on the Demographic and Health Survey 2019, which was 43% (20). This increase could reflect the government's efforts towards universal health coverage (21). In contrast to the current study, in an analysis of the 2016 Ethiopian Demographic and Health Survey, distance to the health centre was associated with four or more antenatal care visits (7). Furthermore, in an analysis of data from Demographic and Health Surveys in five East African counties 2010-2014 showed that geographical accessibility of health facilities had a strong influence on maternal health care utilization (22). The study employed cost-distance analysis based on the health facilities' position and difficulties in traversing the surface of each 300x300 m cell in the study areas. The analysis included the slopes based on elevation measurements as well as the distance from home to the health facility. These findings imply that efforts climbing up and down the hills were barriers to accessing these services. A similar result was shown regarding the influence of household wealth on antenatal care use and facility delivery in the previous study in the same study area (19).

We found that more than half of the study women had delivered at a health facility, which was similar to the coverage (48%) reported by the 2019 Ethiopian Demographic Health Survey (20). In line with the current study, there was also a marked wealth-based inequity in the utilization of antenatal care four or more times and facility delivery in a study performed in the same areas two years earlier (19). Reviews of similar Ethiopian studies (23), research from sub-Saharan Africa countries (24) and East African countries (22), as well as from a range of low-income countries (25) point at considerable regional variation, social inequities in the utilization of facility delivery services, and the importance of topography

and distance from home to health centres and hospitals. A study from Kenya which attempted to disentangle distance to facility and quality of care indicated that pregnant women bypassed possible places to deliver, preferring to give birth at a facility that provided better-quality care (26). Furthermore, an analysis from 81 low and middle-income countries suggested that closing the quality gap would enhance the use of services (27). Such evidence suggest that the problem with distance to the health facility may be less important if the services offered maintain a high quality. In some areas, it may be motivated to consider outreach antenatal clinics organized by the health centres. The problems with distance to facilities for delivery services may be managed by schemes to organize transport or manage associated costs or by improving maternal waiting homes.

In terms of utilization of child health services, thirty-six percent of children aged 12-23 months had received all basic immunizations. This low level was similar to the national estimate (38%) from 2019 Ethiopian mini-Demographic and Health Survey (18). A study two years earlier in the same rural areas as the current study showed an equitable distribution of full child immunization across different socio-economic groups (19). This relative equity in vaccination coverage could be explained by the rural outreach services, organized from the health posts, where eligible children were summoned to vaccination days in their local villages (19). Our analysis showed that the medium tertile of distance to health post was linked to lower immunization coverage and sick child healthcare utilization. This may be a random error, given the lack of consistency across distance strata. If true, it may be a reflection of the geographic distribution of outreach services. A study in Ethiopia's hard-to-reach areas, distance to the health facility influenced full child immunization coverage in addition to the mother's education, utilization antenatal delivery services and child delivered at the health facility (28). However, in an analysis of the 2016 Demographic and Health Survey, full child immunization showed significant variation between groups characterized by geographic, socioeconomic, and maternal factors with pro-advantaged bias (29). The immunizations are given at the health post and partly through outreach services in the villages. This

combination of facility-based and outreach services may theoretically not only increase coverage but also enhance equity (30,31).

One-third of the children 2-59 months, who had been sick in the two weeks before the survey, had sought care from health posts, health centres, hospitals or private clinics which is in line with the national estimates of the utilization of health services for sick children (32). Our results showed no association between care-seeking for sick children and distance to the nearest health centre, difference in altitude and socio-economic status. During the last decade, Ethiopia has scaled up the integrated Community Case Management of common childhood illnesses provided by salaried health extension workers at the health posts (33). The availability of community-level primary care services at health posts close to home, offering child health services, could partly explain the relative equity in utilization. Contrary to this, the 2016 nationally representative Demographic Health Survey showed inequities in care-seeking for common childhood illnesses between groups defined by geographic region, mother's education, or household wealth (32). The Ethiopian government has expanded maternal, neonatal, and child health services in the country (34,35). Further efforts are needed not only to expand these services but also to increase the quality of care (36,37) to reach the goal of Universal Health Coverage (21).

The difference in the influence of distance and difference in altitude between maternal and child health service may be related to the differences in service provision. The child health services, mainly managed by the health posts, include outreach activities with immunization and home-based care. Women had to reach the more distant health centres to receive antenatal and delivery care.

The sample for this study was drawn from intervention and comparison areas for the evaluation of the Optimization of the Health Extension Program intervention, which was implemented in selected districts of the four most populous Ethiopian regions (15). Thus, the results may not be generalizable to the population at country level. We have, however, reasons to believe that the selected districts are typical for agrarian areas of these major Ethiopian regions. Previously published studies have focused on one or a few components of maternal and child care utilization (38,39), but we assessed the major service

coverages, i.e., antenatal care visits, facility delivery, immunization, and utilization of health services for sick children. In cross-sectional studies like this, there is a risk of biased reporting of care utilization. To reduce that risk, we limited the maternal services indicators to the last 12 months. The immunization coverage adhered to the methodology of the Demographic and Health Surveys, including children aged 12-23 months. Similarly, we followed the common practice of limiting common childhood illnesses and care-seeking to a two-weeks recall.

We assessed the straight-line distance between home and the nearest health facility. In reality, it may not be possible to travel in a straight line to a specific location and the route from home to the nearest health facility may be longer than the straight line as one has to avoid obstacles such as a river or a steep slope. In such cases, using cost-distance measurements had given a more realistic measurement. Some home-to-facility distance data were missing, especially regarding the maternal indicators, i.e., the distance from home to the health centre. The reason was missing GPS coordinates of the health centre the family was supposed to use. We have no reason to believe that these omissions introduced any biased estimates. We added the difference in altitude as a potentially important dimension in care utilization. In Ethiopia, with its mountains and valleys, the difference in altitude expresses some of the time and exact distance by road or path from home to the health facility.

We assessed altitude by GPS data that may not always be accurate. However, the error is usually of little importance; in the range from 10 to 20 meters. The socio-economic status was based on assets and characteristics of the household in the way that is practised in the Demographic and Health Surveys. This characterization represents accumulated wealth and resources but does not directly represent the economic strength of the household to cover costs for care-seeking. Given the relative imprecision of the included determinants, we categorized these data into tertiles.

We used multi-level regression modelling that included the determinants, the outcome, and potential confounding factors at the cluster, household, and individual levels. The regression models accounted for confounding and clustering effects.

CONCLUSION

The topography, i.e., the distance and difference in altitude of the areas, and socio-economic status of the household affected the utilization of maternal health services. We did not find such inequities in the utilization of child health services, although coverage was on a low level. The strive for universal health coverage in Ethiopia will require not only increased coverage but also targeted efforts to address the geographic and social differentials in care utilization.

For peer review only

List of abbreviations

AOR	Adjusted Odds Ratio
GPS	Geographical Positioning System
ICC	Intra-class Correlation Coefficient

Declaration

Funding

The study was funded by a grant to the London School of Hygiene & Tropical Medicine from the Bill and Melinda Gates Foundation (OPP1132551)

Availability of data

Data could be available at a reasonable request to the corresponding author, Atkure Defar, atkuredefar@gmail.com

Authors' contributions

The study was conceived by AD with inputs from LÅP and KA. AD, LÅP, KA, ZT and YO contributed in the acquisition, analysis, or interpretation of data. AD prepared the first draft of the manuscript. LÅP, YO, ZT and KA contributed to the interpretation of results and revision of the manuscript. All authors read and commented on the manuscript and approved the final version of the paper.

Consent for publication

Not applicable

Competing interests

None declared

Acknowledgements

The authors would like to thank the University of Gondar for the support provided to the project. We are also grateful to the regional health bureaus and district health offices in the study areas for their smooth

For peer review only

For peer review only - http://bmjopen.bmj.com/site/about/guidelines.xhtml

and kind cooperation. Special thanks to all field staff for their excellent fieldwork and to the study participants for their kind cooperation in interviews and data collection.

REFERENCES

1. World Health Organization, The World Bank. Tracking Universal Health Coverage - First Global Monitoring Report. Geneva, WHO; 2015.

2. Boerma T, Requejo J, Victora CG, Amouzou A, George A, Agyepong I, et al. Countdown to 2030: tracking progress towards universal coverage for reproductive, maternal, newborn, and child health. Lancet [Internet]. 2018 Apr 14;391(10129):1538–48. Available from: https://doi.org/10.1016/S0140-6736(18)30104-1

3. Schaaf H, Hesse G. Schwindelerkrankungen im kindesalter: Erweiterte diagnosemöglichkeiten der vestibulären komponente. Vol. 79, Padiatrische Praxis. 2012. p. 253–65.

4. Memirie ST, Verguet S, Norheim OF, Levin C, Johansson KA. Inequalities in utilization of maternal and child health services in Ethiopia: The role of primary health care Health systems and services in low and middle income settings. BMC Health Serv Res [Internet]. 2016;16(1):1–8. Available from: http://dx.doi.org/10.1186/s12913-016-1296-7

5. Kassile T, Lokina R, Mujinja P, Mmbando BP. Determinants of delay in care seeking among children under five with fever in Dodoma region, central Tanzania: a cross-sectional study. Malar J. 2014 Sep;13:348.

6. Lohela TJ, Campbell OMR, Gabrysch S. Distance to Care, Facility Delivery and Early Neonatal Mortality in Malawi and Zambia. PLoS One. 2012;7(12).

7. Tegegne TK, Chojenta C, Getachew T, Smith R, Loxton D. Antenatal care use in Ethiopia: A spatial and multilevel analysis. BMC Pregnancy Childbirth. 2019;19(1):399.

8. Tadele N, Lamaro T. Utilization of institutional delivery service and associated factors in Bench Maji zone, Southwest Ethiopia: community based, cross sectional study. BMC Health Serv Res. 2017;17(1):1–10.

9. Jimma R, District H, Zegeye K, Gebeyehu A, Melese T. Primary Health Care : Open Access The Role of Geographical Access in the Utilization of Institutional Delivery. Prim Heal care. 2014;4(1):1–6.

10. Defar A, Okwaraji YB, Tigabu Z, Persson LÅ, Alemu K. Geographic differences in maternal and child health care utilization in four Ethiopian regions; a cross-sectional study. Int J Equity Health. 2019;18(1):173.

11. Shiferaw S, Spigt M, Seme A, Amogne A, Skrøvseth S, Desta S, et al. Does proximity of women to facilities with better choice of contraceptives affect their contraceptive utilization in rural Ethiopia? PLoS One. 2017;12(11).

12. Tanou M, Kamiya Y. Assessing the impact of geographical access to health facilities on maternal healthcare utilization: Evidence from the Burkina Faso demographic and health survey 2010. BMC Public Health. 2019;19(1):1–8.

13. Nesbitt RC, Lohela TJ, Soremekun S, Vesel L, Manu A, Okyere E, et al. The influence of distance and quality of care on place of delivery in rural Ghana. Sci Rep [Internet]. 2016;6(August):1–8.

For peer review only

Available from: http://dx.doi.org/10.1038/srep30291

14. Sarrassat S, Meda N, Badolo H, Ouedraogo M, Some H, Cousens S. Distance to care, care seeking and child mortality in rural Burkina Faso: findings from a population-based cross-sectional survey. Trop Med Int Health. 2019 Jan;24(1):31–42.

15. Berhanu D, Okwaraji YB, Belayneh AB, Lemango ET, Agonafer N, Birhanu BG et al. Protocol for the evaluation of a complex intervention aiming at increased utilization of primary child health services in Ethiopia. A before and after study in intervention and comparison areas. BMC Health Serv Res. 2020;20(1):339–12.

16. CSA. Population Census Commission ii [Internet]. Mortality. 2007. Available from: http://www.csa.gov.et/surveys/Population and Housing census/ETH-pop-2007/survey0/data/Doc/Reports/National_Statistical.pdf

17. Central Statistical Agency and ICF International, Findings K. Ethiopia Demographic and Health Survey, 2011 [Internet]. Addis Ababa, Ethiopia; 2011. Available from: Central Statistical Agency (CSA) [Ethiopia] and ICF. 2017. 2016 Ethiopia Demographic and Health Survey Key Findings. Addis Ababa, Ethiopia, and Rockville, Maryland, USA. CSA and ICF

18. Tamirat KS, Sisay MM. Full immunization coverage and its associated factors among children aged 12-23 months in Ethiopia: Further analysis from the 2016 Ethiopia demographic and health survey. BMC Public Health. 2019;19(1):1–7.

19. Wuneh AD, Medhanyie AA, Bezabih AM, Persson LÅ, Schellenberg J, Okwaraji YB. Wealth-based equity in maternal, neonatal, and child health services utilization: A cross-sectional study from Ethiopia. Int J Equity Health. 2019;18(1):1–9.

20. Ethiopian Public Health Institute (EPHI) and ICF. 2019. Mini Demographic and Health Survey 2019: key Indicators. Rockville, Maryland, USA: EPHI and ICF. 2019.

21. Alebachew A, Hatt L, Kukla M. Monitoring and Evaluating Progress towards Universal Health Coverage in Ethiopia. PLoS Med. 2014;11(9):9–11.

22. Ruktanonchai CW, Ruktanonchai NW, Nove A, Lopes S, Pezzulo C, Bosco C, et al. Equality in maternal and newborn health: Modelling geographic disparities in utilisation of care in five East African countries. PLoS One. 2016;11(8):1–17.

23. Kebede A, Hassen K, Teklehaymanot AN. Factors associated with institutional delivery service utilization in Ethiopia. Int J Womens Health. 2016;8:463–75.

24. Moyer CA, Mustafa A. Drivers and deterrents of facility delivery in sub-Saharan Africa: A systematic review. Reprod Health. 2013;10(1):40.

25. Karra M, Fink G, Canning D. Facility distance and child mortality: a multi-country study of health facility access, service utilization, and child health outcomes. Int J Epidemiol. 2017 Jun;46(3):817–26.

26. Escamilla V, Calhoun L, Winston J, Speizer IS. The Role of Distance and Quality on Facility Selection for Maternal and Child Health Services in Urban Kenya. J Urban Health. 2018 Feb;95(1):1–12.

27. Chou VB, Walker N, Kanyangarara M. Estimating the global impact of poor quality of care on

For peer review only

For peer review only - http://bmjopen.bmj.com/site/about/guidelines.xhtml

maternal and neonatal outcomes in 81 low- And middle-income countries: A modeling study. PLoS Med. 2019;16(12):1–16.

28. Girmay A, Dadi AF. Full Immunization Coverage and Associated Factors among Children Aged 12-23 Months in a Hard-to-Reach Areas of Ethiopia. Int J Pediatr. 2019;2019:1–8.

29. Geweniger A, Abbas KM. Childhood vaccination coverage and equity impact in Ethiopia by socioeconomic, geographic, maternal, and child characteristics. Vaccine [Internet]. 2020;38(20):3627–38. Available from: https://doi.org/10.1016/j.vaccine.2020.03.040

30. Oyo-Ita A, Cs W, Oringanje C, Ce N, Oduwole O, Mm M. Interventions for improving coverage of childhood immunisation in low- andmiddle-income countries. Cochrane Database Syst Rev [Internet]. 2016;(7). Available from: https://www.ncbi.nlm.nih.gov/pubmed/27394698

31. Utazi CE, Thorley J, Alegana VA, Ferrari MJ, Takahashi S, Metcalf CJE, et al. Mapping vaccination coverage to explore the effects of delivery mechanisms and inform vaccination strategies. Nat Commun [Internet]. 2019;10(1):1–10. Available from: http://dx.doi.org/10.1038/s41467-019-09611-1

32. Central Statistical Agency (CSA) [Ethiopis], ICF. Ethiopia Demographic and Health Survey 2016. Addis Ababa, Ethiopia, and Rockville, Maryland, USA; 2016.

33. Miller NP, Amouzou A, Tafesse M, Hazel E, Legesse H, Degefie T, et al. Integrated Community Case Management of Childhood Illness in Ethiopia : Implementation Strength and Quality of Care. Am J Trop Med Hyg. 2014;91(2):424–34.

34. Byrne A, Hodge A, Jimenez-Soto E, Morgan A. What works? Strategies to increase reproductive, maternal and child health in difficult to access mountainous locations: A systematic literature review. PLoS One. 2014;9(2).

35. Karim AM, Tamire A, Medhanyie AA, Betemariam W. Changes in equity of maternal, newborn, and child health care practices in 115 districts of rural Ethiopia: Implications for the health extension program. BMC Pregnancy Childbirth [Internet]. 2015;15(1):1–11. Available from: http://dx.doi.org/10.1186/s12884-015-0668-z

36. Allen E, Schellenberg J, Berhanu D, Cousens S, Marchant T. Associations between increased intervention coverage for mothers and newborns and the number and quality of contacts between families and health workers: An analysis of cluster level repeat cross sectional survey data in Ethiopia. PLoS One. 2018;13(8):1–13.

37. Defar A, Getachew T, Taye G, Tadele T, Getnet M, Shumet T, et al. Quality antenatal care services delivery at health facilities of Ethiopia, assessment of the structure/input of care setting. BMC Health Serv Res. 2020;20(1):485.

38. Ayalneh AA, Fetene DM, Lee TJ. Inequalities in health care utilization for common childhood illnesses in Ethiopia: evidence from the 2011 Ethiopian Demographic and Health Survey. Int J Equity Health [Internet]. 2017;16(1):67. Available from: http://equityhealthj.biomedcentral.com/articles/10.1186/s12939-017-0561-7

39. Alene M, Yismaw L, Berelie Y, Kassie B. Health care utilization for common childhood illnesses in rural parts of Ethiopia: Evidence from the 2016 Ethiopian demographic and health survey. BMC Public Health. 2019;19(1):1–12.

For peer review only

For peer review only - http://bmjopen.bmj.com/site/about/guidelines.xhtml

Figure legend

Figure 1 Maps showing A) Ethiopia, B) the four study regions, and C) the 46 study districts in the four regions (Graph constructed in ArcGIS 10.4 software using freely available data from the Ethiopian Central Statistics Agency)

Figure 2: The distribution of antenatal care, facility delivery, child immunization and sick childcare utilization by tertiles of the distance and difference in altitude between the household and the nearest health facility, and the wealth index.

For peer review only

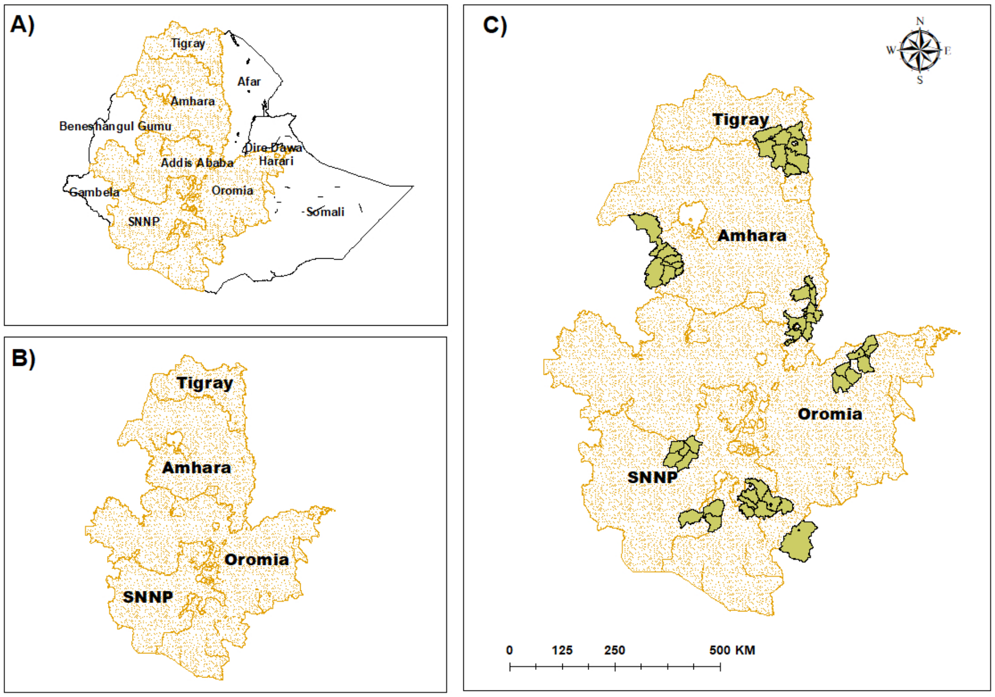

296x210mm (96 x 96 DPI)

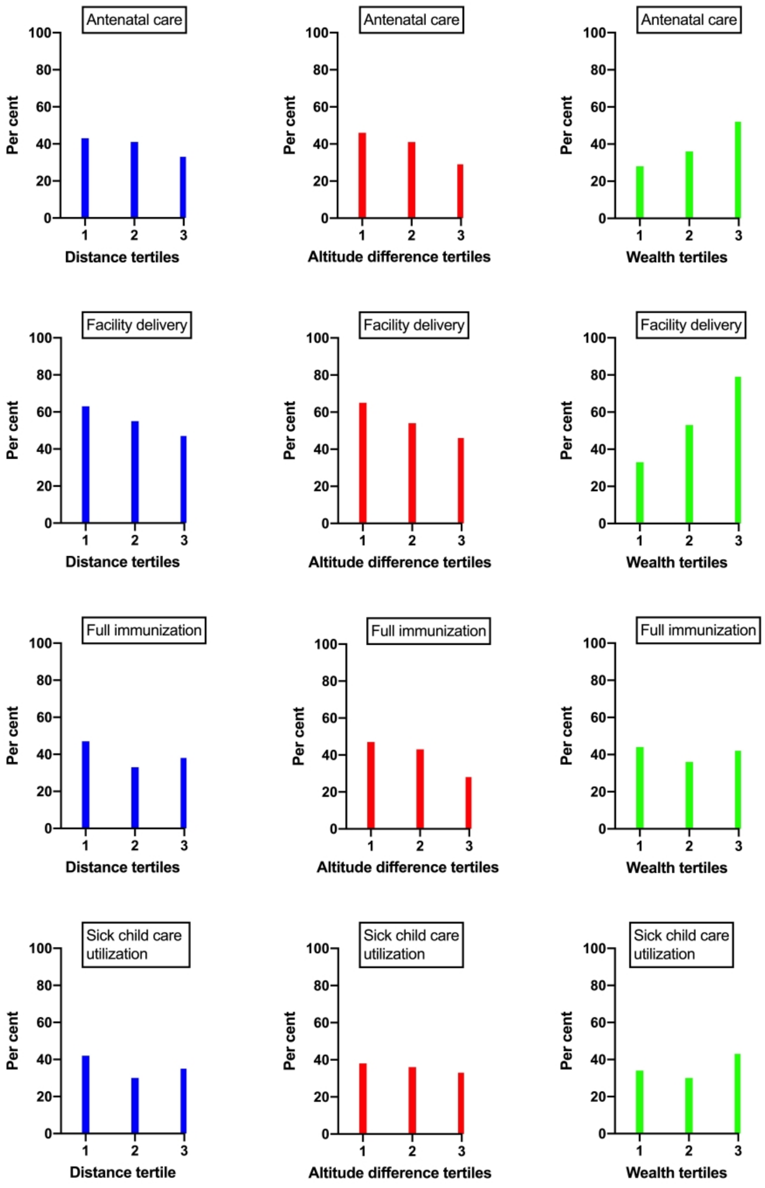

178x269mm (600 x 600 DPI)

STROBE—Checklist of items for a cross-sectional studies

Manuscript section	Item No	Description	Reported ✓	Page #
**Title and abstract**	1	(*a*) Indicate the study's design with a commonly used term in the title or the abstract		
		(*b*) Provide in the abstract an informative and balanced summary of what was done and what was found	✓	2
**Introduction**				
Background/rationale	2	Explain the scientific background and rationale for the investigation being reported	✓	4-5
Objectives	3	State specific objectives, including any prespecified hypotheses	✓	5
**Methods**				
Study design	4	Present key elements of study design early in the paper	✓	6
Setting	5	Describe the setting, locations, and relevant dates, including periods of recruitment, exposure, follow-up, and data collection	✓	6
Participants	6	(*a*) Give the eligibility criteria, and the sources and methods of selection of participants	✓	6-7
Variables	7	Clearly define all outcomes, exposures, predictors, potential confounders, and effect modifiers. Give diagnostic criteria, if applicable	✓	7
Data sources/ measurement	8*	For each variable of interest, give sources of data and details of methods of assessment (measurement). Describe comparability of assessment methods if there is more than one group	✓	*2 & 7*
Bias	9	Describe any efforts to address potential sources of bias	✓	4
Study size	10	Explain how the study size was arrived at		
Quantitative variables	11	Explain how quantitative variables were handled in the analyses. If applicable, describe which groupings were chosen and why	✓	6-7
Statistical methods	12	(*a*) Describe all statistical methods, including those used to control for confounding	✓	8
		(*b*) Describe any methods used to examine subgroups and interactions		
		(*c*) Explain how missing data were addressed	✓	8
		(*d*) If applicable, describe analytical methods taking account of sampling strategy	✓	8
		(*e*) Describe any sensitivity analyses		
**Results**				
Participants	13*	(a) Report numbers of individuals at each stage of study—eg numbers potentially eligible, examined for eligibility, confirmed eligible, included in the study, completing follow-up, and analysed	✓	9-10
		(b) Give reasons for non-participation at each stage		

For peer review only

		(c) Consider use of a flow diagram		
Descriptive data	14*	(a) Give characteristics of study participants (eg demographic, clinical, social) and information on exposures and potential confounders	✓	10
		(b) Indicate number of participants with missing data for each variable of interest	✓	10
Outcome data	15*	Report numbers of outcome events or summary measures	✓	10
Main results	16	(*a*) Give unadjusted estimates and, if applicable, confounder-adjusted estimates and their precision (eg, 95% confidence interval). Make clear which confounders were adjusted for and why they were included	✓	11
		(*b*) Report category boundaries when continuous variables were categorized	✓	10
		(*c*) If relevant, consider translating estimates of relative risk into absolute risk for a meaningful time period		
Other analyses	17	Report other analyses done—eg analyses of subgroups and interactions, and sensitivity analyses		
**Discussion**				
Key results	18	Summarise key results with reference to study objectives	✓	12-13
Limitations	19	Discuss limitations of the study, taking into account sources of potential bias or imprecision. Discuss both direction and magnitude of any potential bias	✓	4
Interpretation	20	Give a cautious overall interpretation of results considering objectives, limitations, multiplicity of analyses, results from similar studies, and other relevant evidence	✓	12-14
Generalisability	21	Discuss the generalisability (external validity) of the study results	✓	14
**Other information**				
Funding	22	Give the source of funding and the role of the funders for the present study and, if applicable, for the original study on which the present article is based	✓	16

*Give information separately for exposed and unexposed groups.

Note: An Explanation and Elaboration article discusses each checklist item and gives methodological background and published examples of transparent reporting. The STROBE checklist is best used in conjunction with this article (freely available on the Web sites of PLoS Medicine at http://www.plosmedicine.org/, Annals of Internal Medicine at http://www.annals.org/, and Epidemiology at http://www.epidem.com/). Information on the STROBE Initiative is available at www.strobe-statement.org.

For peer review only
